# Induced Pluripotent Stem Cells in Non-Model Species: Applications and Challenges

**DOI:** 10.3390/cells15171565

**Published:** 2026-08-28

**Authors:** Qiuye Bao, Nicole Liling Tay, Christina Yingyan Lim, Shangzhe Xie, Soon Chye Ng, Oz Pomp

**Affiliations:** 1 Mandai Nature, 80 Mandai Lake Road, Singapore 729826, Singapore; qiuye.bao@mandai.org.sg (Q.B.); nicole.tay@mandai.org.sg (N.L.T.); christina.lim@mandai.org.sg (C.Y.L.); 2 Mandai Wildlife Group, 80 Mandai Lake Road, Singapore 729826, Singapore; shangzhe.xie@mandai.com; 3 Sincere Healthcare Group, 8 Sinaran Drive, Singapore 307470, Singapore; sc.ng@shg.com.sg

**Keywords:** induced pluripotent stem cells, non-model species, wildlife biobanking, wildlife conservation, one medicine

## Abstract

Induced pluripotent stem cells have revolutionized biomedical research—yet the vast majority of life on Earth remains beyond their reach. Non-model species lack the annotated genomes, validated reagents, and species-specific culture infrastructure that make iPSC technology routine in humans and mice, and this infrastructure deficit, compounded by genuine biological differences in pluripotency network architecture across taxa, is what has kept the field narrow. The deep conservation of the core pluripotency network across vertebrates suggests that reprogramming may, in principle, be achievable across a far broader range of species than currently demonstrated—though the extent to which this holds across more divergent taxa remains to be established. This review consolidates current progress and future potential of iPSC technology across five domains: technical reprogramming challenges and advances; conservation applications including genetic rescue, in vitro gametogenesis, and de-extinction; medical applications within a one medicine framework; agricultural applications spanning disease resistance, climate resilience, and cultured meat; and species-specific iPSC-derived systems in ecotoxicology. Throughout, we distinguish what has been demonstrated from what remains aspirational and identify the priorities that will determine whether the iPSC revolution can be extended—rigorously and at scale—beyond model organism research.

## 1. Introduction

Induced pluripotent stem cells (iPSCs), first generated by Yamanaka and colleagues using the OSKM factors (Oct3/4, Sox2, Klf4, c-Myc), have transformed our understanding of cellular plasticity and opened unprecedented avenues for regenerative biology [1,2]. The foundational insight—that a terminally differentiated somatic cell can be reprogrammed to a pluripotent state capable of giving rise to any cell type of the adult organism—has proven remarkably general. While most iPSC research has focused on humans and classical model species such as the mouse, rat, and non-human primates, a growing frontier lies in the extension of reprogramming technologies to non-model species (NMS): here defined as organisms lacking extensive genetic resources, standardized husbandry protocols, or established stem cell lines. These include companion and livestock species not established in laboratory research, non-traditional laboratory animals such as reptiles, amphibians beyond *Xenopus*, and fish beyond zebrafish, and most wildlife species. Excluded from this definition are the classical laboratory model organisms—mouse, rat, *zebrafish*, *Xenopus*, *Drosophila*, and *C. elegans*—and human, for which extensive iPSC infrastructure already exists. Species such as pig, cattle, and horse occupy a border position: where their iPSC platforms have been developed primarily in an agricultural or biomedical context, they appear in this review as informative comparators or because their platforms are directly relevant to applications in less-studied species; where they are referenced in conservation or ecotoxicological contexts, they function more clearly as NMS.

The case for extending iPSC technology to NMS is not merely scientific curiosity. The accelerating biodiversity crisis, the emergence of zoonotic pathogens at the human–animal–environment interface, the need for more resilient and sustainable livestock systems, and the inadequacy of current ecotoxicological models to represent the physiological diversity of wild species all converge on a common need: biological tools that can operate at the level of the species, not just the standard laboratory model [3]. iPSCs, by enabling the derivation of any cell type from any individual of any species—in principle—offer exactly this. A somatic cell collected from a critically threatened animal, a bushmeat species under poaching pressure, or a sentinel species accumulating industrial contaminants can, once reprogrammed, become an indefinitely expandable, genetically intact cellular resource amenable to differentiation, editing, cryopreservation, and functional interrogation [4].

Recent workshop reports have highlighted the potential of iPSC technology for conservation biology specifically [5], but no existing review synthesizes NMS iPSC applications across the full range of domains examined here—from technical reprogramming advances through veterinary medicine, agriculture, and ecotoxicology—or subjects de-extinction claims to systematic analytical scrutiny.

The relatively high conservation of the core pluripotency network across vertebrates suggests that reprogramming may, in principle, be achievable across a far broader range of species than currently demonstrated [6]. What has primarily limited the field is infrastructure—the absence of annotated reference genomes, validated species-specific reagents, standardized culture conditions, agreed quality control frameworks, and the dedicated funding mechanisms that would make systematic protocol development feasible at scale—though species-specific biological differences in pluripotency signaling, factor compatibility, and epigenetic architecture present genuine additional challenges that are distinct from these infrastructure gaps and require dedicated experimental resolution alongside them. This infrastructure deficit manifests differently across application domains—as culture incompatibility in conservation settings, as differentiation protocol gaps in veterinary medicine, as regulatory uncertainty in agricultural biotechnology—but its root cause is consistent. Resolving it is the central practical challenge facing the field, and the one around which this review is organized.

Despite these constraints, progress has been substantial. From the first proof-of-concept iPSC lines derived from a drill and a Northern white rhinoceros in 2011 [7], the field has expanded to encompass megafauna, marsupials, monotremes, primates, birds, bats, and early forays into fish [8]. Non-integrative reprogramming methods have matured to the point where genomically intact lines are routinely achievable in mammalian NMS [9]. Primordial germ cell-like cells have been induced from rhinoceros iPSCs. Organoids have been generated from Sumatran rhinoceros cells [10]. CRISPR-based genome editing has identified host factors governing African swine fever virus replication in porcine iPSC-derived macrophages [11]. CRISPR/Cas9 introduction of the feline HCM-causing MYBPC3/R820W mutation into human iPSC-derived cardiomyocytes has recapitulated hypertrophic cardiomyopathy at the cellular level, demonstrating the translational value of iPSC platforms for studying species-specific cardiac disease [12]. Cultured meat companies are leveraging bovine and porcine iPSC platforms as scalable cell sources [13]. Each of these achievements represents not only a species-specific technical milestone but a proof of principle that the iPSC framework is extensible—that the biological logic developed in mouse and human can, with appropriate adaptation, be made to work across the diversity of vertebrate life.

This review consolidates current progress and future potential of iPSCs derived from NMS across five domains. Section 2 examines the technical challenges specific to NMS reprogramming—pluripotency state instability, genomic and reagent limitations, functional validation gaps—and the expanding toolkit of factor combinations and non-integrative delivery methods developed in response, closing with a call for agreed minimum reporting standards and strategic species prioritisation. Section 3 addresses conservation applications, tracing the shift from integrating to integration-free approaches across threatened mammals and birds, and situating iPSC technology within the broader genetic rescue toolkit alongside biobanking, somatic cell nuclear transfer, and assisted reproductive technologies including in vitro gametogenesis and germline applications. It also examines de-extinction, where iPSCs serve as the multiplex-editable cellular platform for proxy species generation, with critical attention to the definitional and governance questions raised by recent high-profile projects.

**Figure 1:**
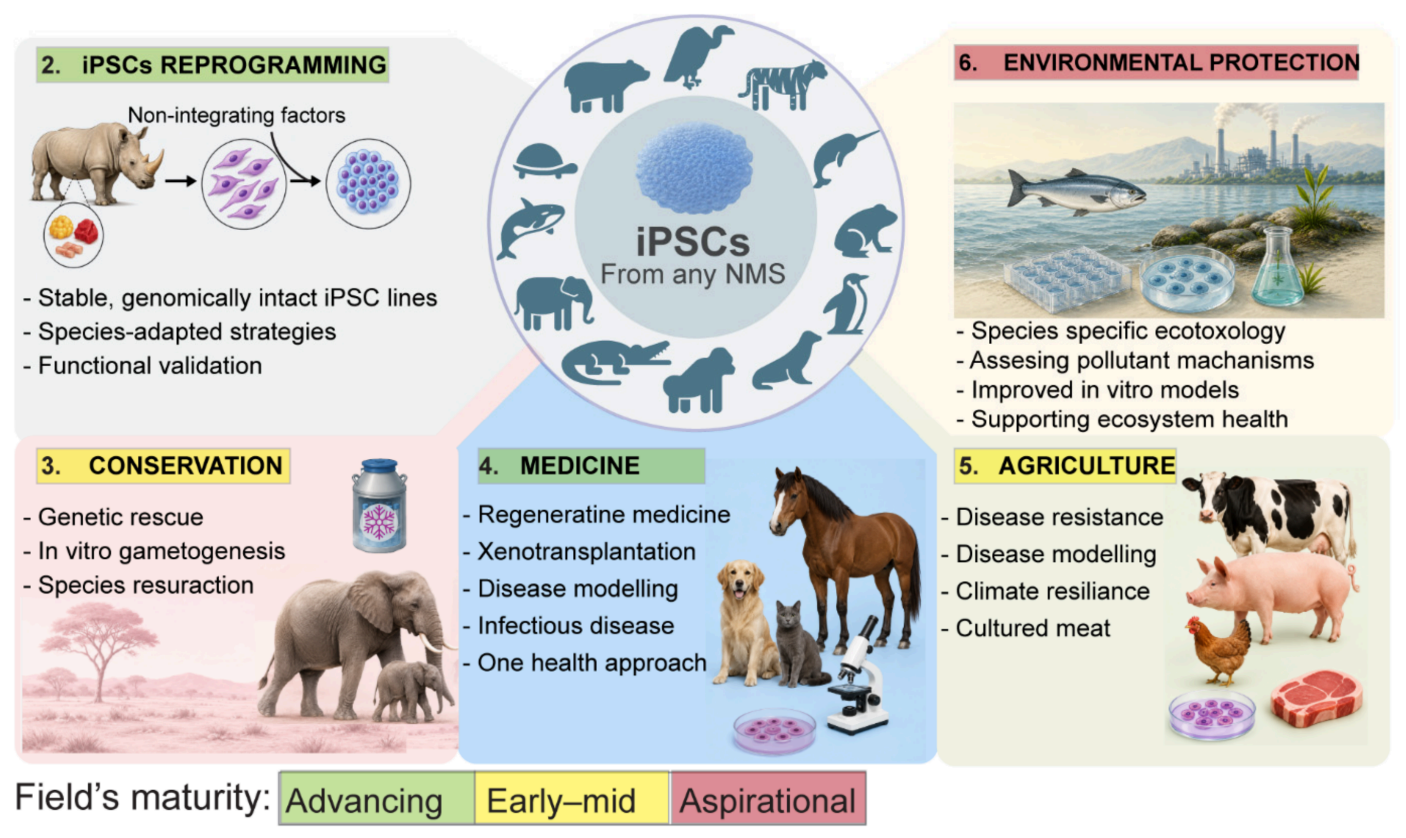
Conceptual framework of the review. Schematic overview positioning iPSC technology as a central enabling platform that underpins the technical foundation (Section 2), encompasses the derivation of stable, genomically intact iPSC lines using non-integrating delivery systems and species-adapted reprogramming strategies, together with functional validation and the need for agreed minimum standards. From this foundation extend four application domains: conservation (Section 3); medicine (Section 4); agriculture (Section 5); and environmental protection (Section 6). Section numbers correspond to the review structure.

Section 4 turns to medicine, reviewing iPSC-based regenerative applications and xenotransplantation, disease modelling within a one medicine framework, infectious disease platforms for host-restricted pathogens, and zoonotic surveillance—set against the backdrop of the first regulatory approvals of iPSC-derived therapies in Japan and advancing clinical trials in the United States. Section 5 addresses agricultural applications spanning disease resistance engineering, climate resilience, and cultured meat. Section 6 makes the case for species-specific iPSC-derived systems in ecotoxicology, with an honest assessment of what is currently actionable versus aspirational. Throughout, we critically examine the technical, ecological, and regulatory considerations that will determine whether the iPSC revolution can be meaningfully extended beyond the traditional boundaries of model organism research. Honest communication of stage distinctions is also a scientific priority: conflating iPSC derivation with gamete production, or gamete production with population recovery, risks both inflated public expectations and misallocated research investment. The overall framework of this review is summarized in Figure 1 and Table 1.

Several emerging applications of NMS iPSC technology fall outside the scope of this review but merit explicit acknowledgement. Organoid technology—encompassing intestinal, airway, liver, kidney, reproductive tract, and brain organoids derived from NMS iPSCs—has the potential to serve as a unifying platform across conservation, medicine, and ecotoxicology, and warrants dedicated treatment as the field matures. Comparative developmental biology and evolutionary medicine represent a particularly compelling future domain: iPSCs from species with exceptional longevity (naked mole rat, bowhead whale), cancer resistance (elephant, shark), or physiological extremes (hibernating mammals, diving cetaceans) offer a route to interrogating adaptive mechanisms without embryos from protected animals. Reproductive biology beyond in vitro gametogenesis—including gonadal organoid systems, folliculogenesis, and spermatogenesis—similarly remains an underdeveloped but high-priority frontier. Comparative immunology, functional genomics enabled by CRISPR screens and single-cell atlases, and the extension of ecotoxicological frameworks to natural toxins, microplastics, and nanoparticles are additional areas where NMS iPSC platforms are positioned to make distinctive contributions. These directions are noted here to map the field’s expanding horizon rather than its current boundaries.

## 2. Technical Challenges and Advances in iPSC Reprogramming for Non-Model Species

### 2.1. Background

The generation of iPSCs from non-model species (NMS) presents challenges that are qualitatively distinct from routine derivation in humans or mice. Unlike in these well-characterised systems—where optimised factor combinations, validated culture conditions, and robust quality control frameworks are firmly established—reprogramming in NMS must contend with fundamental biological unknowns, infrastructural deficits, and species-specific barriers that compound at every stage of the workflow. Understanding these challenges is a prerequisite for the rational design of solutions, and the field has begun to respond with an expanding toolkit of adapted methodologies. Before examining these challenges in detail, it is necessary to establish two areas of background knowledge essential for interpreting what follows: the nature of pluripotency states across vertebrates, and the distinction between integrating and non-integrative reprogramming approaches.

Pluripotency exists not as a single state but as a continuum—naïve, formative, and primed—each with distinct signalling dependencies, epigenetic configurations, and developmental potentials [14,15]. Naïve cells require LIF/STAT3 and dual GSK3/MEK inhibition (2i/LIF); primed cells depend on FGF and Activin; formative cells occupy a transitional intermediate competent to respond to germ-layer inductive cues. This distinction is particularly consequential for NMS: the optimal pluripotency state for most non-model species is unknown, standard 2i/LIF and FGF/Activin conditions routinely fail to sustain NMS iPSCs, and the absence of single-cell preimplantation reference data for most taxa means that reprogramming outcomes cannot be benchmarked against any in vivo standard.

Reprogramming approaches are distinguished by whether transgenes become permanently incorporated into the host genome—a distinction developed in detail in Section 2.3.2, and introduced here only to orient the reader. Integrating methods offer high transduction efficiency but carry risks of insertional mutagenesis and genomic instability that render them unsuitable for translational, reproductive, or germline applications. Non-integrating approaches generate genomically intact, footprint-free iPSCs and are strongly preferred wherever downstream fidelity is required, though generally at lower efficiency and greater technical demand. The choice between approaches is therefore not merely technical but consequential for the downstream utility of any line established.

### 2.2. Challenges

The following three subsections identify the primary biological, molecular, and conceptual obstacles to iPSC generation in NMS. Together they define the problem space that the methodological advances in Section 2.3 are designed to address.

#### 2.2.1. Pluripotency State Instability and Culture Incompatibility

A primary obstacle in NMS reprogramming is pluripotency state instability. Reprogrammed cells from NMS frequently default to intermediate or poorly defined states that resist long-term maintenance under standard conditions. This is not simply a culture optimization problem: it reflects genuine biological uncertainty about which pluripotency state—naïve, formative, or primed—is the natural ground state for a given species, and whether the states characterized in mouse and human have direct equivalents across vertebrate diversity [14,15]. The two-inhibitor (2i/LIF) conditions that stabilise naïve murine pluripotency, and the FGF/Activin conditions that support primed human ESCs, routinely fail to sustain NMS iPSCs, producing either spontaneous differentiation or growth arrest rather than stable self-renewal [16]. Commercial maintenance media such as mTeSR—optimized for human iPSCs and incorporating undefined animal-derived components—introduce a further layer of uncertainty when applied to novel species, compounding the difficulty of interpreting failed cultures.

The problem is sharpened by the relative scarcity of single-cell embryogenesis data for most NMS. In mouse and human, comparative transcriptomic atlases of preimplantation development now provide a high-resolution map of the molecular transitions through which naïve, formative, and primed states are traversed in vivo [17]. For the vast majority of NMS, no such reference exists, meaning that the target pluripotency state cannot be empirically defined and reprogramming outcomes cannot be benchmarked against an in vivo standard.

Compounding this, for a novel NMS with no established protocol, each of the following culture variables must also be treated as empirically unknown rather than transferable from model species. Feeder-cell choice—mouse embryonic fibroblasts, immortalised MEFs, species-matched feeders, or feeder-free systems [18,19]—and coating substrate—Matrigel, vitronectin, or specific laminin isoforms such as laminin-511 and laminin-521 [20,21]—each influence attachment, survival, and self-renewal, yet the combination that supports a given NMS cannot be predicted in advance. Feeder choice carries an additional molecular dimension beyond matrix mechanics: because feeders act in part by secreting paracrine factors such as LIF, FGF2, and TGF-β1 [22,23], a species-mismatched feeder layer may fail for cross-species signaling reasons—a distinct problem from matrix incompatibility, addressed separately in Section 2.2.4. Additional variables compound the search space: basal medium formulation and serum or serum-replacement source; oxygen tension [24]; passaging method and its interaction with dissociation-induced apoptosis [25]; seeding density; and the concentration and timing of small-molecule pathway modulators [26]. Any of these, individually or in combination, can convert an otherwise competent reprogramming attempt into apparent failure, and none can be assumed to transfer from a model species.

The practical consequence is that, for a novel NMS with no established protocol, sequential single-variable optimization is prohibitively slow and consumes scarce and often irreplaceable primary material. A more tractable strategy is to screen multiple conditions in parallel from the outset—varying, for example, pluripotency-state regime (2i/LIF versus FGF/Activin versus intermediate formulations), feeder and matrix, and oxygen tension across a factorial or matrix design—so that the permissive region of the condition space is identified empirically rather than assumed. Where primary cell numbers are severely limiting, miniaturized or microwell-format screens allow a broader condition set to be surveyed from a fixed starting population. This exploratory posture—treating the correct conditions as unknown and to be discovered rather than inherited from model-species protocols—is, at present, the most realistic route to establishing stable lines in species for which no reference protocol exists.

#### 2.2.2. Genomic and Molecular Limitations

Incomplete genome assemblies constitute a second major constraint. For species lacking high-quality reference genomes, the identification of pluripotency gene orthologs is imprecise, promoter analyses to guide transgene design are unreliable, and cross-species transcriptomic comparisons—essential for evaluating reprogramming fidelity—are compromised [27,28]. The problem extends to reagent availability: validated antibodies against pluripotency markers such as OCT4, SOX2, NANOG, and SSEA antigens have been developed and quality-controlled for human and mouse epitopes, and cross-reactivity in NMS is inconsistent and frequently unreported. Immunofluorescence and flow cytometric characterization of putative iPSC colonies—standard in human and murine systems—therefore cannot be assumed to be reliable in novel species without independent antibody validation.

Karyotypic instability represents an additional genomic concern. Even when stable iPSC lines are established, chromosomal abnormalities arise at elevated frequency during prolonged culture, and transgene silencing efficiency—critical for confirming that reprogramming factor expression has been extinguished and that the self-renewal program is genuinely endogenous—remains unpredictable across species [29]. Without reliable silencing, the risk persists that apparent iPSC maintenance reflects ongoing transgene activity rather than stable pluripotency.

Beyond confounding pluripotency assessment, unsilenced reprogramming transgenes actively oppose lineage commitment during directed differentiation—constitutive expression of OCT4, SOX2, KLF4, or c-MYC resists the transcriptional remodeling required for germ layer specification, meaning that transgene-active lines frequently fail at the differentiation stage regardless of their apparent pluripotency characteristics.

#### 2.2.3. Functional Validation Challenges

A deeper conceptual problem concerns the benchmarks used to validate iPSC identity. In mice, the gold standard for genuine pluripotency is germline-competent chimera formation: injection of iPSCs into blastocysts, with subsequent contribution to all somatic tissues and the germline of the resulting animal. This benchmark has been achieved for only a handful of NMS—most notably rabbit [30]—and remains undemonstrated for the overwhelming majority of species for which iPSC generation has been claimed [8]. In its absence, published NMS iPSC lines are typically validated by surrogate criteria: alkaline phosphatase activity, pluripotency marker expression, embryoid body formation, and teratoma assays. While these are meaningful indicators, they are insufficient to exclude the possibility that cells represent partially reprogrammed intermediates rather than bona fide pluripotent stem cells. The field would benefit from agreed minimum validation standards for NMS iPSCs; lines with incomplete evidence should be described as putative iPSC-like cells rather than bona fide iPSCs.

#### 2.2.4. Cross-Species Incompatibility of Reprogramming Reagents

A final challenge specific to NMS, and one that connects several of the barriers above, is that the reprogramming and maintenance toolkit was optimized almost entirely in human and murine systems and cannot be assumed to transfer seamlessly to divergent taxa. Incompatibility can arise at three mechanistically distinct levels: the secreted ligand–receptor interface, the intracellular reprogramming-factor interface, and the innate sensing of the delivery vehicle. Distinguishing these matters, because each fails differently and each is addressed by a different intervention.

At the level of secreted factors, incompatibility is less pervasive than first principles would predict. The core self-renewal signaling modules—FGF, TGF-β/Activin/Nodal, and the gp130–JAK–STAT3 axis—are deeply conserved across vertebrates, and recombinant human FGF2 and Activin A support pluripotency across a broad taxonomic range, including avian and non-primate mammalian systems in which the ligand–receptor interface remains functional despite considerable evolutionary distance [31,32,33,34]. The principal exception is leukemia inhibitory factor (LIF), whose failure to sustain NMS iPSCs was noted in Section 2.2.1. LIF signaling illustrates why sequence conservation is a poor predictor of cross-species function: despite approximately 78% ligand and 76% receptor identity between mouse and human, mouse LIF binds only its cognate receptor whereas human LIF binds both, the species-specificity residing in the receptor’s immunoglobulin-like domain [35,36]. Whether this promiscuity extends across more divergent taxa remains to be systematically characterized; the available experimental evidence covers a limited number of species, and cross-reactivity should not be assumed beyond the systems in which it has been directly tested. In the domestic cat, only feline LIF—not the murine protein conventionally used in rodent systems—maintains iPSC pluripotency [37], a direct demonstration that a reagent validated in model species can fail outright in a non-model one and require a species-specific ortholog. Even where a species-specific factor is not strictly required, apparent LIF failure in a non-model line more often reflects pluripotency-state preference than a recognition defect: most non-rodent species examined to date are more readily stabilized in primed-type, FGF2/Activin-dependent conditions in which LIF–STAT3 signaling does not sustain self-renewal irrespective of whether the ligand is recognized. This distinction between recognition and functional response is frequently collapsed in the literature and should be resolved empirically, using the diagnostic readouts described at the end of this subsection, rather than inferred from colony outcome.

At the intracellular level, the reprogramming factors have proven more tolerant of cross-species transfer than phylogenetic distance would predict, at least in the species examined to date. In the avian species examined to date, pluripotency-associated factors show functional divergence from the mammalian network, including differences involving POU5F3/POUV, SOX-family factors, and KLF-family factors [38]. Yet avian iPSCs have been derived using human or murine reprogramming cocktails despite more than 300 million years of divergence from mammals, indicating that the exogenous mammalian factors engage sufficiently conserved transcriptional targets to initiate reprogramming [38,39]. Incompatibility at this level is therefore quantitative rather than absolute. The canonical mammalian four factors activate part of the endogenous avian network—chicken and duck fibroblasts upregulate endogenous POU5F3 and KLF4—but stall short of full reprogramming, failing to upregulate NANOG and LIN28A and retaining a tumorigenic propensity, until NANOG and LIN28 are supplied exogenously [40]. The appropriate response is therefore not a different class of protein but species-tailored factor identity, stoichiometry, and supplementation, discussed as an advance in Section 2.3.

The third level, innate sensing, concerns the delivery vehicle rather than any maintenance factor or reprogramming protein. Commercial Sendai virus vectors used for reprogramming are specifically engineered—through mutations in the P and L proteins—to minimise innate immune responses, and there is no strong evidence that type I interferon induction is a primary cause of poor transduction efficiency in NMS cells [41,42]. Where Sendai virus performs poorly in a novel NMS cell type, the more likely explanation is the absence of host factors required for viral entry or replication—a tropism constraint rather than an immune barrier. This interpretation is based on mechanistic reasoning rather than direct comparative experimental evidence across NMS taxa, since systematic data on Sendai virus tropism variation across divergent species remain limited. A second and underappreciated limitation of standard commercial Sendai virus vectors is their temperature-sensitive design: the mutations introduced to reduce immunogenicity and facilitate vector clearance also reduce viral persistence and limit the duration and level of transgene expression at physiological temperature, which can be insufficient to drive full reprogramming in cell types that reprogram slowly or require prolonged factor exposure [41]. Taken together, the likely bottlenecks for Sendai virus-based reprogramming in NMS cells are tropism incompatibility, inefficient viral replication in divergent host cell environments, and transient vector activity caused by temperature-sensitive mutations—rather than innate immune sensing per se. An alternative non-integrative platform with broader species tropism is Venezuelan equine encephalitis virus (VEEV)-based replicons, which can deliver reprogramming factors across a wider range of cell types, though here innate immune sensing is more important and requires active suppression using B18R, a vaccinia virus-derived decoy receptor for type I interferons [43]. Moving forward, a more effective strategy for NMS reprogramming may be to use persistent Sendai virus vectors lacking temperature-sensitive mutations—maintaining sustained transgene expression throughout the reprogramming window—combined with siRNA targeting of viral genes for controlled vector elimination once reprogramming is complete, and species-specific reprogramming factor combinations as discussed in Section 2.3.1 [42]. A cell line that responds poorly to standard temperature-sensitive Sendai virus should therefore prompt consideration of persistent vector formats or alternative delivery platforms rather than immediate attribution of failure to the pluripotency network or culture conditions.

Two practical points follow for non-model derivation. First, reagent incompatibility should not be assumed to be the dominant cause of reprogramming failure without pathway-level evidence to support it. Premature transgene silencing, incomplete epigenetic resetting, karyotypic instability, and culture adaptation each produce the same terminal phenotype—absence of stable colonies—and are readily misattributed to reagent non-recognition when the underlying mechanism is not interrogated. The exceptions are instructive rather than refuting: feline LIF represents a case where species-specific substitution was strictly required, and in avian species, NANOG and LIN28 supplementation identifies a genuine reagent-level bottleneck that mammalian OSKM alone cannot resolve [40]. Critically, both failures were identified because pathway-level readouts were applied—without them, the outcome would have been indistinguishable from culture failure or epigenetic stalling. Second, and following directly from this: cleanly attributing a reprogramming failure to reagent incompatibility requires a receptor-engagement or pathway-activation readout—ligand-induced phosphorylation of STAT3, SMAD2/3, or ERK, for example—rather than inference from colony morphology or count alone. In the absence of such evidence, the conclusion that a given human factor or standard maintenance medium has failed in an NMS context should be treated as a working hypothesis requiring mechanistic resolution—not as an established finding that justifies protocol switching. Substituting a species-specific ortholog, changing the growth medium, or adding supplementary factors without first identifying the level at which the current approach fails risks compounding unknown variables and forfeiting the diagnostic clarity that pathway-level readouts would otherwise provide. The primary challenges identified across Section 2.2, and the methodological responses developed in Section 2.3, are summarized in Table 2.

### 2.3. Advances & Responses

The following three subsections describe the methodological toolkit that has emerged in response to the challenges above—spanning factor composition, delivery method, and quality standards—and assess how far each advance has moved the field toward reliable, broadly applicable NMS reprogramming (Table 1).

#### 2.3.1. Expanded and Species-Specific Factor Combinations

The first methodological response to NMS reprogramming challenges concerns factor composition. Canonical OSKM combinations, optimised through iterative screening in mouse and human, frequently perform poorly in NMS—not because the pluripotency network is absent but because the transcriptional wiring that OSKM engages is species-specifically configured. Three complementary strategies have emerged in response: substitution of canonical factors with species-appropriate orthologues, supplementation with auxiliary factors that address species-specific network gaps, and engineering of enhanced reprogramming factors with amplified transcriptional potency.

The relationship between factor origin and reprogramming outcome is species-specific rather than following a simple rule of homology, and the evidence base now spans multiple taxa with instructively varied outcomes. The porcine case is the most systematically documented in livestock: early piPSC lines generated with human or murine OSKM consistently failed to silence exogenous transgenes and showed insufficient activation of endogenous pluripotency markers, whereas porcine-origin OSKM activated Hippo, PI3K-Akt, and MAPK signalling pathways to levels resembling the porcine blastocyst inner cell mass [44]. In felids and canines, species-derived factors produced more efficient reprogramming than their human counterparts: in cats, feline LIF—rather than the murine or human protein—is strictly required for iPSC maintenance, a case of absolute species-specificity rather than a quantitative efficiency difference [37,45]; and in dogs, a custom Sendai virus vector encoding six canine factors (OSKMLN) produced more efficient reprogramming than human counterparts, with complete transgene silencing achieved under feeder-free conditions [46].

The divergence from the mammalian pluripotency blueprint is the most structurally profound of any vertebrate group examined to date: avian ESC biology indicates that birds encode POU5F3 rather than POU5F1 as the functional OCT4 equivalent, express SOX3 rather than SOX2 as the effective SoxB1 factor, and deploy KLF3, KLF5, or KLF6 rather than KLF4—substitutions that reflect genuine evolutionary divergence in the transcriptional architecture of the pluripotency network rather than simple sequence drift [47,48]. Whether systematically replacing mammalian OSKM components with these avian-appropriate orthologues improves reprogramming efficiency beyond what has been achieved with mammalian factors plus NANOG and LIN28 supplementation remains an open and important experimental question—one that is now technically tractable given the availability of annotated avian genomes and established avian iPSC lines as validation benchmarks.

#### 2.3.2. Auxiliary Factor Supplementation

Where canonical OSKM engages an incomplete version of the endogenous pluripotency network, supplementary factors can bridge the gap. In avian cells, NANOG and LIN28 supplementation is required to advance reprogramming beyond the partially reprogrammed state reached by mammalian OSKM alone—chicken and duck fibroblasts upregulate endogenous POU5F3 and KLF4 under mammalian OSKM but stall without NANOG and LIN28, retaining tumorigenic propensity until these are supplied [40]. In felids and bovidae, NANOG, LIN28, and the histone demethylase KDM4A have been found to enhance or complement OSKM components, with factor requirements varying substantially across taxa [49,50]. Notably, two recent studies, currently under review, demonstrated that human pluripotency factors can reprogram porcine cells into bona fide iPSCs when combined with appropriate culture optimisation [51,52], suggesting that factor identity and protocol optimisation interact—and that species-specific factors are not always strictly required when culture conditions adequately support the endogenous network.

#### 2.3.3. Engineered Super-Factors

Beyond substitution and supplementation, a parallel strategy involves engineering reprogramming factors themselves to be more potent transcriptional activators. The most significant advance in this direction is the development of enhanced SOX factors—principally SOX17 fusions and the so-called Super-SOX constructs—which carry amplified transactivation domains that dramatically increase reprogramming efficiency in human cells and, critically, in species where endogenous SOX engagement is partial [53,54,55]. SOX constructs, generated by fusing the SOX2 HMG domain with a potent transactivation domain derived from VPR or similar synthetic activators, achieves reprogramming in human cells with reduced factor combinations—in some formulations replacing the entire OSKM cocktail—and shows enhanced performance in cell types that are refractory to conventional OSKM, including aged cells and cells from species with divergent SOX network wiring [56,57]. Analogous engineering strategies have been developed for OCT4 and KLF4, producing enhanced POU (ePOU) and KLF (eKLF) constructs that address reduced chromatin accessibility and ortholog divergence respectively, and which represent prospective tools for NMS species where these factors are the primary rate-limiting step. In the NMS context, the principle is directly applicable: species in which endogenous SOX2 or its ortholog is poorly engaged by the exogenous human factor are strong candidates for Super-SOX-based approaches. The MyoD transactivation domain fusion strategy—in which reprogramming factors are fused to the MyoD transactivation domain to increase chromatin accessibility at pluripotency loci—represents a related engineering approach that has improved reprogramming efficiency in porcine and bovine cells [58,59]. Chemical enhancement of transcription factor activity through small molecules targeting chromatin remodelling—VPA, sodium butyrate, BIX-01294—provides a pharmacological parallel to factor engineering that is particularly relevant for NMS where vector payload constraints limit the number of transgenes deliverable simultaneously.

#### 2.3.4. Key Success Stories

**Figure 2:**
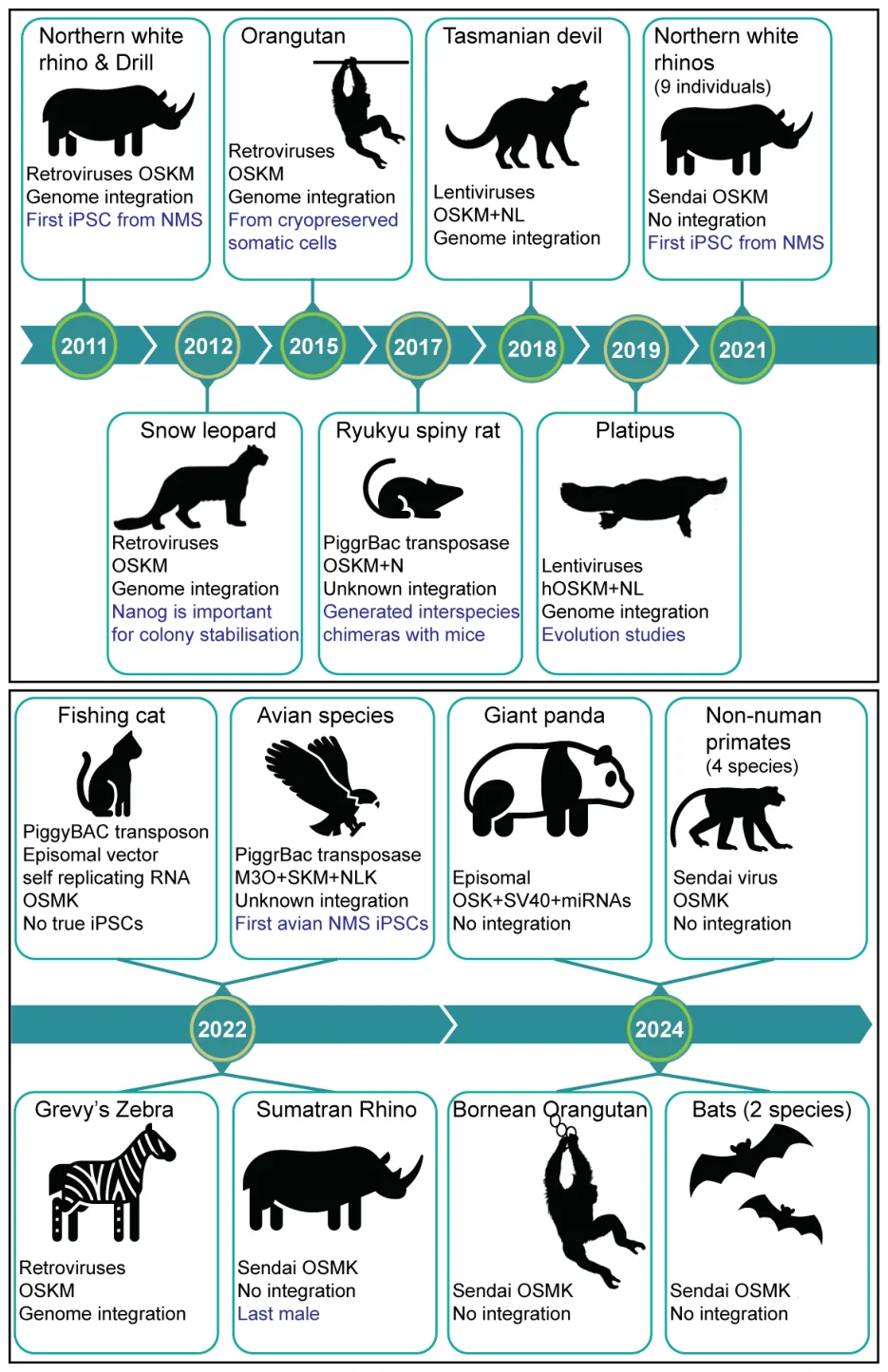
Timeline of iPSC derivation milestones across non-model species, 2011–2024. Selected first or otherwise significant reprogramming achievements are arranged chronologically, illustrating both the widening taxonomic range of non-model species iPSC work and the progressive shift in methodology over time. NMS include: Northern white rhino (*Ceratotherium simum cottoni*) and Drill (*Mandrillus leucophaeus*) [7], Snow leopard (*Panthera uncia*) [65], orangutan (*Pongo pygmaeus*) [66], Ryukyu spiny rat (*Tokudaia osimensis*) [67], Tasmanian devil (*Sarcophilus harrisii*) [68], Platypus (*Ornithorhynchus anatinus*) [69], Northern white rhinoceros (*Ceratotherium simum cottoni*) [9], Fishing cat (*Prionailurus viverrinus*) [70], Threatened Avian species [60], Grevy’s zebra (*Equus grevyi*) [71], Sumatran rhinoceros (*Dicerorhinus sumatrensis*) [10], Giant panda (*Ailuropoda melanoleuca*) [61], Non-human primates [72], Bornean orangutan (*Pongo pygmaeus*) [73], Bats [74]. For each entry, the panel indicates the species, the reprogramming factor combination and delivery vector, the resulting transgene integration status, and the primary reference. The sequence traces the field from early integrative approaches—retroviral and lentiviral delivery of the canonical reprogramming factors, which dominated the first derivations—toward non-integrative strategies including Sendai virus, episomal vectors, and self-replicating RNA in more recent work. Abbreviations: OSKM, OCT4/SOX2/KLF4/c-MYC; N, NANOG; L, LIN28; SV40LT, Simian virus 40 Large T antigen; M3O, modified OCT4, created by fusing the mammalian *Oct3/4* gene to the transactivation domain (TAD) of the *MyoD* protein; miRNAs, micro RNAs 302–367 cluster containing microRNAs: miR- 302b, miR- 302c, miR- 302a, miR- 302d, and miR- 367. See also Supple Table S1.

Several milestones illustrate the cumulative impact of these approaches. First, the derivation of avian iPSCs from four endangered Japanese species—Okinawa rail, ptarmigan, fish owl, and golden eagle—using a PiggyBac system delivering seven mouse factors including NANOG and LIN28 represented the first demonstration of iPSC derivation from birds, achieved only after the factor combination was extended beyond canonical OSKM [60]. Second, the derivation of giant panda iPSCs using a fully chemically defined approach combining OCT4, SOX2, and KLF4 with the iCD3 small molecule cocktail achieved reprogramming without animal-derived components—a significant advance for a critically endangered species where reagent control and genomic fidelity are paramount [61]. Third, the generation of iPSCs from nine individuals representing the last northern white rhinoceros individuals using Sendai virus, enabling PGCLC induction and the establishment of an allelic diversity resource from a functionally extinct species, represents the clearest demonstration that non-integrative reprogramming with appropriately chosen factors can achieve conservation-grade cellular rescue from the brink of extinction [9] (Figure 2). It is also worth noting the preprint showing the derivation of Bornean orangutan iPSCs using Sendai virus reprogramming from fibroblasts, with resulting cells exhibiting primed state pluripotency characteristics which can be upgraded to naïve state with PGCLC potential by aggregation, demonstrated that delivery method and culture condition optimisation together can achieve a pluripotency state in a great ape that recapitulates the most developmentally useful form of pluripotency known in any species [62]. Taken together, these success stories share a common feature: each required deliberate species-specific adaptation of the factor combination, delivery method, or culture condition rather than direct application of human protocols—a consistent finding that supports the principle that wherever annotated genomes permit the cloning of native orthologs or the characterization of species-specific network topology, this information should be used to guide factor design from the outset rather than treated as a secondary optimization step. An important caveat, however, is that annotated genome quality cannot be assumed: in the canine system, the NCBI-predicted KLF4 sequence contained errors that were only identified and corrected through experimental validation by PCR in primary dog cells—a finding that underscores the need to verify computational predictions empirically before designing NMS reprogramming factors [46].

#### 2.3.5. Non-Integrative Delivery Methods

Integrating methods—principally retroviral and lentiviral vectors—dominated early NMS reprogramming and retain practical relevance for contexts where efficiency is the primary constraint and genomic fidelity is not. Retroviruses transduce dividing cells with high efficiency and undergo transcriptional silencing upon successful reprogramming; when starting material is limited, lentiviruses extend this to non-dividing or slowly dividing primary cells, which is advantageous when starting material is [1,63]. The liabilities are well-documented: stable genomic integration carries risk of insertional mutagenesis, incomplete silencing may sustain residual transgene expression that confounds pluripotency assessment, and integrated cassettes render cells unsuitable for translational, reproductive, or germline applications [49]. For basic research where the goal is simply to demonstrate reprogramming feasibility in a novel species, integrating vectors remain a pragmatic first-pass approach—but any line intended for downstream conservation, clinical, or agricultural use should be derived by non-integrative means from the outset.

For such applications, the shift to non-integrative delivery is not merely preferable but essential: cells intended for assisted reproduction, embryo generation, or germline use must be genomically intact, and insertional mutagenesis renders them unsuitable regardless of pluripotency characteristics [49]. Three non-integrative platforms have emerged as leading options for NMS, alongside the intermediate excisable transposon approach.

Sendai virus—a negative-sense RNA virus that replicates exclusively in the cytoplasm without nuclear entry—has become the most widely adopted non-integrative system across mammalian NMS, combining high transduction efficiency with a clean genomic footprint and commercial availability [64]. Its adoption has driven the recent expansion of integration-free iPSC generation across rhinoceros, primates, pigs, and bat species. Its principal limitation is that it was developed and optimised for human cells, and transduction efficiency in morphologically or physiologically distinct cell types from divergent species can be substantially lower.

Episomal vectors—self-replicating DNA plasmids progressively lost as cells divide—offer an alternative with a smaller technical footprint, requiring only standard transfection equipment rather than biosafety level 2 viral work. Episomal approaches have successfully generated iPSCs from giant panda and several Southeast Asian primate species [61,72], and their practical accessibility makes them particularly relevant for institutions without viral vector infrastructure. Their principal limitation is lower reprogramming efficiency relative to Sendai virus, which may be prohibitive when starting material is scarce.

Self-replicating RNA (saRNA) vectors represent an emerging non-integrative alternative that addresses one of the principal limitations of conventional synthetic mRNA delivery: the requirement for high input doses to achieve sustained transgene expression [75]. saRNA constructs encode both the reprogramming factors and a viral RNA-dependent RNA polymerase (typically derived from alphaviruses such as Venezuelan equine encephalitis virus) that amplifies the RNA intracellularly [76], producing high and prolonged transgene expression from a substantially lower initial dose without nuclear entry or genomic integration [43]. This amplification property is particularly relevant for NMS reprogramming, where transfection efficiency into primary somatic cells is often poor [77], starting material is scarce, and repeated transfection—the standard workaround for conventional mRNA delivery [78]—may not be feasible. saRNA-based reprogramming has been demonstrated in human cells and selected NMS [43], and while the approach remains less widely adopted than Sendai virus or episomal vectors, its low-dose, footprint-free profile positions it as a strong candidate for resource-limited conservation settings in which biosafety level 2 viral infrastructure is unavailable and starting cell numbers are low.

The excisable piggyBac transposon system occupies an intermediate position: it integrates into the genome during reprogramming but can theoretically be removed by transposase-mediated excision, generating transgene-free iPSCs at efficiencies comparable to viral methods [79]. It has been employed successfully in Ryukyu spiny rat and avian NMS [60,67]. Excision is not always precise, however, and residual mutations at excision sites have been documented [60,80,81], so the system should not be treated as equivalent to fully non-integrative methods in applications where absolute genomic fidelity is required.

Fully chemically defined reprogramming—avoiding both viral vectors and animal-derived components entirely—represents the most stringent approach and has been achieved in a small number of NMS, including giant panda iPSCs generated using OCT4, SOX2, and KLF4 alongside miRNA plasmids and the iCD3 small molecule cocktail [61], and porcine iPSCs under a defined medium incorporating CHIR99021, IWR-1, WH-4-023, LIF, Activin A, and FGF2 [82]. These examples also illustrate the importance of culture condition and media optimization as an independent advance alongside factor and vector choice: the iCD3 system eliminated serum and undefined paracrine components entirely, and the porcine formulation stabilized a pluripotency state not achievable under standard mTeSR or 2i/LIF conditions—demonstrating that medium composition can be the rate-limiting variable in NMS reprogramming independent of factor or delivery method (see Section 2.2.1 for the corresponding challenges). The trajectory of NMS iPSC derivation across species and methods is summarized in Figure 2 and Supplementary Table S1, which includes for each species the cell source, reprogramming method and factors used, integration status, pluripotency validation assays reported, differentiation demonstrated, and major application domain.

#### 2.3.6. Minimum Standards and Prioritization

Collectively, the technical challenges described above make clear that iPSC generation in NMS cannot yet be treated as a routine procedure. Each new species encountered is, in a meaningful sense, a new problem requiring bespoke solutions at the level of factor choice, delivery method, culture conditions, and validation strategy. Two practical responses to this reality are worth emphasising.

First, the field requires agreed minimum reporting and validation standards for NMS iPSC claims—specifying which pluripotency markers, functional assays, and genomic quality control steps constitute an acceptable evidence base for a published iPSC line. The ISSCR guidelines for stem cell research and clinical translation provide a framework applicable to human and model species iPSCs [83], but no equivalent standard has been formally adopted for NMS, and the heterogeneous validation depth evident across the published literature reflects this gap directly. We propose that, in the absence of germline-competent chimera formation—which cannot be required for most NMS given logistical and ethical constraints—a minimum evidence base for classifying a cell line as a bona fide NMS iPSC should comprise five elements: (1) pluripotency marker expression confirmed at both transcript and protein level for the species-appropriate orthologues of the core pluripotency factors—OCT4/POU5F1 or POU5F3 as well as SOX2 or SOX3 depending on taxon, and NANOG—with independent antibody cross-reactivity validation or orthogonal RNA-seq confirmation given the inconsistent cross-reactivity of commercial antibodies raised against human or murine epitopes; where species-specific antibodies are unavailable, transcript-level confirmation by RNA-seq mapped to a species-appropriate reference is acceptable as the primary evidence; (2) transgene silencing confirmation by quantitative RT-PCR using exogenous-specific primers distinguishing endogenous from vector-derived transcripts; (3) functional pluripotency demonstrated by spontaneous or directed differentiation into derivatives of all three germ layers, by embryoid body formation with lineage marker confirmation, or by teratoma assay where feasible and ethically permissible; (4) genomic integrity confirmed by G-banding karyotyping at a defined early passage (P5–10), with copy number variation assessment where resources permit; and (5) stable self-renewal for a minimum of ten passages under defined conditions without spontaneous differentiation or morphological instability. Lines meeting all five criteria may be described as bona fide iPSCs; lines meeting fewer should be described as putative iPSC-like cells or partially reprogrammed intermediates. These criteria are proposed as a minimum achievable standard calibrated to NMS realities, modelled on the ISSCR guidelines [83] but adapted to the practical constraints of the field.

Second, given the resource intensity of species-specific protocol development, strategic prioritisation is essential: investment should be concentrated on species that are simultaneously conservation-critical, technically tractable, and capable of generating broadly transferable methodological insights. As genomic resources improve, organoid technology matures, and non-integrative reprogramming efficiency increases across taxa, the per-species cost of iPSC derivation will decline—but in the near term, selectivity is a scientific and practical necessity.

#### 2.3.7. Applications of iPSCs in Human and Model Species: The Baseline from Which NMS Work Departs

The trajectory toward clinical deployment has accelerated markedly: as of December 2024, 115 regulatory-approved trials were underway testing 83 hPSC-derived products in over 1200 patients, with no generalised safety concerns reported [84]. In February 2026, Japan granted conditional marketing authorisation to the world’s first two iPSC-derived therapies—ReHeart (iPSC-derived cardiomyocyte sheets for heart failure; Cuorips) and AMCHEPRY (Sumitomo Pharma 2026)—while BlueRock Therapeutics received FDA IND clearance for OpCT-001, an iPSC-derived photoreceptor therapy entering Phase I/IIa evaluation (BlueRock Therapeutics). Also, autologous or HLA-matched allogeneic iPSC-derived cell products have entered early-phase trials for conditions including age-related macular degeneration, Parkinson’s disease, heart failure, and cancer therapy, with demonstrated feasibility and initial safety profiles [85,86,87,88,89]. Complementing human studies, iPSCs derived from model species—including mouse, rat, pig, and non-human primates—serve as indispensable preclinical platforms enabling evaluation of graft safety, biodistribution, teratoma formation, immune compatibility, and long-term functional integration in immunocompetent, physiologically relevant whole-organism systems prior to human application [90,91].

Taken together, these milestones confirm that human iPSC-derived cell therapies have crossed from experimental biology into regulated clinical and commercial practice, establishing the safety, manufacturing, and regulatory precedent that NMS applications will eventually need to meet.

## 3. Conservation Applications

### 3.1. iPSCs as a Versatile Tool for Wildlife Genetic Rescue

The accelerating extinction and biodiversity crisis has pushed traditional conservation methods—habitat protection, anti-poaching patrols, and captive breeding—to their limits [8]. In response, a suite of biotechnologies, collectively termed genetic rescue, has emerged as a potentially transformative complement to these established approaches.

Biobanking has become a vital pillar of genetic rescue. Facilities such as the Frozen Zoo at the San Diego Zoo Wildlife Alliance store tissues, somatic cells, gametes, and embryos from thousands of individuals across hundreds of species, providing the raw biological material for downstream reproductive and biotechnological applications including Somatic Cell Nuclear Transfer (SCNT), iPSC derivation, and Assisted Reproductive Technologies (ART). The logic is straightforward: even where a species has suffered a severe genetic bottleneck, its preserved living cells and genetic material remain available for future population supplementation and diversity recovery. ART—including artificial insemination, in vitro fertilisation (IVF), and intracytoplasmic sperm injection (ICSI)—represents the most established application of such biobanked material, and in several species has already contributed to population recovery efforts.

Interspecies SCNT (iSCNT) circumvents the reliance on oocytes from threatened species by using those from closely related, more accessible surrogates. However, the approach is beset by compounding limitations. Efficiency is low even between closely matched species and has been confirmed in only a small number of species combinations—among them gaur cloned using domestic cattle oocytes and African wildcats using domestic cat oocytes—with failures far outnumbering successes across the broader range of attempts [92]. The use of heterologous oocytes introduces additional complications, including mitochondrial DNA incompatibility and failure of embryonic genome activation, rendering the process an unreliable pathway for most threatened species [93]. Mitochondrial-nuclear incompatibility may present a species-specific barrier even within closely related taxa: our laboratory’s work using iPSCs from endangered Suidae species has identified potential mitochondrial transspecies incompatibility as a constraint on surrogate selection within this family, suggesting that phylogenetic proximity alone is insufficient to predict iSCNT permissiveness and that surrogate selection requires empirical validation at the mitochondrial-nuclear interface [94]. Beyond these biological constraints, iSCNT is logistically demanding and costly: it requires access to recipient oocytes and surrogate mothers that are themselves non-threatened, reproductively tractable, and available in sufficient numbers—conditions that are difficult to meet for most wildlife species, which lack a domesticated or otherwise accessible close relative that can serve in this role. Domestic cattle, pigs, and rabbits have been deployed as universal surrogate oocyte donors in some studies, but phylogenetic distance compounds already poor nuclear-cytoplasmic reprogramming efficiency, and the procedural costs—spanning hormone-stimulated oocyte retrieval, nuclear transfer, embryo culture, and surgical embryo transfer—collectively render iSCNT an impractical route for broad application in wildlife conservation.

iPSC technology reframes rather than eliminates these challenges, offering a complementary route that bypasses the most constraining dependencies of the cloning pipeline. Somatic cells from a threatened species can be reprogrammed into iPSCs using non-integrating methods, expanded indefinitely in culture, cryopreserved, and later differentiated into functional gametes or even blastoids—synthetic embryo-like structures capable of mimicking embryo development [8]. Critically, because iPSC lines can be established from many individuals, including those that died without reproducing, they are inherently more genetically representative than SCNT-based approaches, which are constrained by the availability of oocytes and reproductive surrogates. The derivation of iPSCs from nine genetically distinct Northern white rhinoceros individuals—several of whom never reproduced in life—illustrates this advantage directly, capturing allelic diversity that would otherwise have been permanently lost [9]. It is important to note that iPSC-based gamete derivation and blastoid generation remain largely proof-of-concept in most NMS, and the path from banked somatic cells to a live animal involves substantial unresolved biological and technical steps. Nevertheless, iPSCs establish a uniquely flexible starting point: a living, expandable, genomically intact cellular resource that can be revisited as the reproductive technologies required to deploy it continue to mature.

### 3.2. In Vitro Gametogenesis and Germline Applications

One of the most consequential long-term applications of iPSC technology in conservation is in vitro gametogenesis (IVG): the derivation of functional sperm and eggs from pluripotent stem cells [95,96,97]. IVG would enable gamete generation from deceased, sterile, or otherwise non-reproductive individuals, with the resulting gametes deployed through ART to restore genetic diversity to otherwise unrecoverable populations. The stakes are highest for species such as the Northern white rhinoceros, where only two non-reproductive females survive, and the window for conventional reproductive rescue has effectively closed [9,98].

The foundational proof-of-concept for iPSC-based IVG was established in the mouse. Hayashi and colleagues demonstrated that both spermatogonial stem cells and functional oocytes could be derived from mouse ESCs and iPSCs via an epiblast-like intermediate state, with the resulting gametes producing fertile offspring upon fertilisation and embryo transfer [95,99]. A fully in vitro pipeline—requiring no transplantation into a living animal at any stage—was subsequently achieved for oogenesis in mouse, in which PGCLCs were cocultured with reconstituted ovarian somatic cells to produce metaphase II oocytes capable of fertilization [97]. These murine milestones established the conceptual and technical framework upon which conservation-focused IVG efforts are now being built. A critical insight from this work is that PGCLCs alone are insufficient: functional gametogenesis requires the support of gonadal somatic cell environments—either through transplantation or in vitro reconstitution—that provide the niche signals governing meiotic entry, epigenetic reprogramming, and sex-specific maturation [96]. Developing equivalent gonadal somatic cell systems for non-rodent species represents one of the field’s most pressing unresolved challenges.

Progress in translating IVG to conservation-relevant NMS has been meaningful but incremental. The most significant advance to date was the robust induction of PGCLCs from both Southern and Northern white rhinoceros ESCs and iPSCs by the BioRescue consortium [100]. This was the first time PGCLCs had been generated from a large, threatened mammalian species, and required identification of SOX17—rather than the murine PRDM14—as the key transcriptional driver of rhinoceros PGC induction, underscoring that germline specification pathways are not fully conserved across mammals and must be characterised de novo in each taxon. Surface markers CD9 and ITGA6 were identified as conserved across both rhinoceros subspecies, providing tools for PGCLCs isolation applicable to future gamete maturation steps. These PGCLCs now represent the last remaining biological bridge between the nine cryobanked Northern white rhinoceros iPSC lines and the production of functional gametes—a pipeline that, if completed, would constitute the first recovery of a functionally extinct species through iPSC-derived reproduction.

It is worth situating iPSC-based IVG within the broader NWR recovery strategy, since it complements rather than replaces existing approaches. The most realistic near-term pathway to a live NWR calf is conventional IVF/embryo transfer: the BioRescue consortium has already produced NWR blastocysts by ICSI of cryopreserved NWR semen into oocytes aspirated from the two surviving females under ultrasound-guided transvaginal collection, and transfer into southern white rhinoceros surrogates is the immediate next clinical step—all components are established or in active development [3]. The IVF route is, however, constrained by oocyte yield from two aging, non-reproductive females. iPSC-based IVG addresses this directly: by unlocking the genomes of the ten additional banked individuals from whom only somatic cells—not gametes—were collected, IVG would dramatically expand the allelic diversity accessible for reproduction and transform the programme from a two-individual to a fourteen-individual genetic base. Cloning via iSCNT, while theoretically feasible given the availability of banked somatic cells, is not a primary strategy: iSCNT replicates single genomes without recombination and therefore cannot restore population genetic diversity, only replicate what already exists. The overarching constraint on NWR population recovery, however, is not reproductive technology but habitat: no combination of IVF, IVG, or cloning can restore a wild population without secured protected range in the species’ historical territory in Central Africa—a political and conservation challenge that lies entirely outside the scope of reproductive biology.

Beyond rhinoceros, proof-of-concept germline progress has been achieved across several taxa. Female Ryukyu spiny rat (*Tokudaia osimensis*) iPSCs produced spermatocytes and spermatids when injected into male mouse testes—a remarkable finding given the species’ unusual XO/XO sex chromosome system, which lacks a Y chromosome entirely, and one that demonstrated the flexibility of the germline specification pathway across divergent reproductive architectures [60,67]. In rhesus macaque, iPSCs were differentiated into functional spermatogonial stem cells that, upon transplantation into sterilised testes, restored spermatogenesis—providing a non-human primate model with direct relevance to both conservation and reproductive medicine [101]. PGCLCs have been generated from cynomolgus monkey ESCs and iPSCs under WNT-inhibited defined conditions, with transcriptomic profiles closely resembling embryonic PGCs in vivo [102]. For the common marmoset, an mRNA-based induction protocol generated PGCLCs and subsequent gonocyte-like cells, advancing understanding of primate germline specification in a species increasingly used as a bridge model between rodents and humans [103].

Complementing iPSC-based approaches, spermatogonial stem cells xenotransplantation strategies—in which testicular tissue biobanked from deceased animals is grafted into reproductively active recipients of a related species to restore spermatogenesis—are under active development as part of integrated conservation germline pipelines [104]. These approaches are not mutually exclusive with IVG: testicular tissue biobanking provides an immediate bridge to germline preservation while longer-term iPSC-based gametogenesis protocols are refined.

Despite this progress, the distance between current achievements and the deployment of IVG-derived gametes in conservation breeding programmes remains substantial. In vitro spermatogenesis beyond the spermatid stage—the step required to generate motile, fertilisation-competent spermatozoa without testicular transplantation—has not been achieved in any species outside the mouse [105]. Full in vitro oogenesis beyond the PGCLCs stage has similarly not been demonstrated in any NMS. Both processes depend critically on the reconstitution of species-specific gonadal somatic environments whose cellular composition, signalling requirements, and developmental timing remain largely uncharacterised outside rodents and a small number of primate species.

The most recent advances in the human IVG baseline illustrate how the gonadal somatic niche bottleneck is beginning to be approached from both sides of gametogenesis. On the female side, Conception reported in June 2026 the first generation of fully iPSC-derived human follicles containing primary oocytes that have entered meiosis, assembled within a niche reconstructed entirely from iPSC-derived granulosa-like cells (The first early human eggs from stem cells—Conception|Advancing the Future of Fertility). On the male side, human and rhesus iPSCs have been differentiated into undifferentiated and differentiated spermatogonia and preleptotene spermatocytes using xenogeneic reconstituted testes assembled from mouse fetal testicular cells—the furthest advance in primate male IVG yet reported [106]. Neither has achieved gametes capable of fertilisation—the Conception oocytes remain at the primordial rather than mature MII stage, separated from fertilisation competence by the entire process of folliculogenesis and oocyte maturation; and Whelan et al. reach spermatogonia and preleptotene spermatocytes, which are progenitor cells rather than fertilisation-competent spermatozoa, with meiotic completion and sperm maturation entirely undemonstrated. The gap between these milestones and a live birth—let alone a self-sustaining population—spans multiple unresolved biological stages, each of which constitutes an independent research frontier. A further consideration, not yet systematically addressed in the conservation context, concerns the fidelity of ART-derived genomes. Large-scale whole-genome sequencing studies in human ART-conceived individuals have identified hints of elevated de novo mutation rates associated with IVF and ICSI procedures [107]. If analogous effects occur in conservation ART—including iPSC-derived IVG pipelines, where cells undergo extensive culture and epigenetic resetting before contributing to the germline—this raises an ethical tension between the goal of resurrecting genomically intact individuals and the pragmatic conservation priority of preserving rare individuals and their genetic diversity, even if they carry minor non-lethal de novo mutations. This tension is not unique to iPSC-based approaches—it applies to all ART in conservation—but it is amplified in IVG pipelines where the number of cell divisions between somatic cell and functional gamete is substantially greater than in conventional reproduction. Acknowledging this uncertainty and building genomic quality control into IVG-derived embryo assessment protocols should be a priority as the field advances.

The field is best understood not as delivering an imminent reproductive solution but as building a staged technical infrastructure across at least five distinct biological transitions—cell line establishment, gamete progenitor induction, gamete maturation, fertilisation and embryo development, and live birth—of which only the first has been achieved in NMS, and the last four remain entirely unresolved. Progress at one stage does not predict or accelerate progress at the next.

### 3.3. De-Extinction and Genome Editing: iPSCs as a Platform for Species Resurrection

De-extinction is not a single, well-defined outcome but a spectrum of biological interventions that differ fundamentally in their ambition, feasibility, and scientific validity. At one end lies functional trait restoration: the introduction of specific adaptive traits from an extinct species into a living relative, producing an animal that can perform an ecological role previously filled by the extinct taxon without recapitulating its full genome or phenotype. In the middle sits the hybrid proxy: an organism whose genome has been edited at many loci with the explicit goal of systematically approximating the extinct species, sharing a substantial proportion of its distinctive biology while remaining genetically a modified version of its living relative. At the far end is true resurrection: the recovery of a reproductively isolated population that is genomically, phenotypically, and ecologically equivalent to the extinct species—an outcome that would require not only a complete and accurate reference genome but also resolved reproductive pipelines, species-authentic developmental environments, and ultimately a self-sustaining wild population. These categories are not merely semantic. They differ in what counts as success, in the scientific rigour with which claims can be evaluated, and in the ecological and ethical frameworks they invoke. iPSC technology plays a distinct role at each position on this spectrum, as one of several editing platforms available for functional trait restoration, as the preferred multiplex-editable substrate for hybrid proxy generation where the scale of editing exceeds what zygote-based approaches can efficiently achieve, and—in the specific case of species for which viable cells were biobanked before extinction—as the reprogrammed cellular vehicle for true resurrection, through which a genomically authentic nucleus can be maintained, expanded, and deployed in iSCNT or IVG pipelines. The distance between current iPSC-based achievements and each endpoint varies enormously and conflating them produces both inflated claims and misplaced scepticism.

A critical biological constraint governs the entire spectrum and is rarely stated explicitly: true resurrection is only achievable if the intact genome of the extinct species is physically accessible in a viable cellular form—which means biobanking is not merely a conservation tool but a prerequisite for keeping true resurrection within the realm of biological possibility. Two routes exist. The first is cryopreserved gametes—sperm amenable to ICSI, or oocytes amenable to IVF—which provide direct access to the intact haploid genome without reprogramming. The second is cryopreserved somatic cells, which can be reprogrammed into iPSCs and deployed either as nuclear donors in iSCNT or as the starting material for IVG pipelines in which differentiation toward PGCLCs reconstitutes the germline from a diploid somatic source. Both routes presuppose that biobanking preceded extinction—and both are closed permanently once it has not. Any strategy that begins instead from the genome of a living relative produces an animal that is fundamentally the living relative’s genome with modifications—it cannot be the extinct species because the starting material is not the extinct species. Ancient DNA provides a parts list, not a working cell: sequence information alone cannot reconstitute the epigenetic architecture, chromatin organisation, and cellular machinery required to develop a viable organism, none of which a reconstructed sequence can supply. This means that for every fully extinct species currently targeted by de-extinction programmes—woolly mammoth, thylacine, dire wolf, dodo, moa—true resurrection is biologically precluded by the same cause: no viable cells were preserved before extinction. The realistic ceiling of any programme working from reconstructed sequence and a living relative’s cells is, by definition, not true resurrection—because the starting material determines the biological category of the outcome, not the ambition of the programme, the number of loci edited, or the language used to describe it. The sole existing exception is the northern white rhinoceros: a functionally rather than fully extinct species whose somatic cells were biobanked from living individuals before the last breeding males died, enabling iPSC derivation from a genomically authentic source and keeping true resurrection biologically possible [9]. For every species currently in decline, the decision of whether to bank today is, in effect, a decision about whether true resurrection remains an option in the future.

It is important to be explicit about what each of these milestones does and does not represent. Generating a stable iPSC line is not equivalent to generating gametes; generating PGCLCs is not equivalent to generating functional oocytes or spermatozoa; generating gametes in vitro is not equivalent to producing a viable embryo; producing an embryo is not equivalent to achieving a live birth; and achieving a single live birth is not equivalent to restoring a self-sustaining population. Each transition represents an independent biological and technical challenge, several of which remain unresolved in any mammalian species outside the mouse, and none of which has been demonstrated in any NMS. The milestones described in this section are genuine and significant—but they are early steps in a pipeline whose later stages are substantially further from realisation than the early stages might suggest.

Against this constraint, the five de-extinction programmes currently underway at Colossal Biosciences—targeting the woolly mammoth, Tasmanian tiger, dire wolf, dodo, and, announced in July 2025 in partnership with Peter Jackson and the Ngāi Tahu Research Centre, the South Island giant moa (*Dinornis robustus*)—are best understood not as exceptions to the rule but as illustrations of it. Each follows the same logic: ancient DNA is recovered from museum or fossil material, divergent loci are identified by comparison with the closest living relative, and CRISPR-based edits are introduced into that relative’s genome via a species-specific cellular platform. Evaluated against the spectrum, each programme occupies a distinct position.

The dire wolf sits firmly at functional trait restoration: the three grey wolves (*Canis lupus*) born in April 2025 carry 20 genomic edits across 14 genes selected for morphological resemblance—coat colour, texture, and body size proxies—with no systematic attempt to approximate the full genomic divergence between *Aenocyon dirus* and its living relative across their 5.7 million years of independent evolution [108]. What matters is not the number of edits but their intent: traits selected for visual resemblance rather than systematic genomic approximation place this programme unambiguously at the functional trait restoration end, regardless of how it is publicly framed.

The woolly mammoth programme is best characterised as pursuing hybrid proxy as its medium-term goal, with functional trait restoration as its near-term deliverable. Asian elephant iPSCs serve as the expandable, multiplex-editable substrate for stacking cold-adaptive edits across haemoglobin oxygen affinity, subcutaneous fat deposition, fur density, and sebaceous gland activity (demonstrated at cellular level) [109], with proof-of-concept multiplex editing of seven genes in mouse zygotes validating the approach (demonstrated in mouse, not elephant) [110]. The intent is systematic genomic approximation of mammoth cold-adaptation biology—placing it in the hybrid proxy category even at its current early stage.

The Tasmanian tiger programme similarly targets hybrid proxy: the goal is broad genomic approximation of thylacine biology in the fat-tailed dunnart (*Sminthopsis crassicaudata*), underpinned by a chromosome-scale genome assembly estimated at more than 99.9% accuracy [111]. Dunnart iPSC derivation amenable to multiplex editing remains the critical and currently unresolved cellular bottleneck (proposed; not yet demonstrated)—without it, the programme cannot advance beyond genomic characterization.

The dodo and moa programmes are at the earliest stages and are best categorized as functional trait restoration in their current form. The dodo programme has achieved Nicobar pigeon PGC culture as a key milestone toward a chimeric surrogate strategy (demonstrated in Nicobar pigeon; chimeric surrogate production proposed); the moa programme has begun ancient genome sequencing across nine species using tinamou or emu PGCs as the editing platform. Neither programme has yet defined a comprehensive editing target set that would qualify as hybrid proxy ambition. The moa programme faces an additional reproductive engineering challenge with no equivalent in mammalian de-extinction: a South Island Giant Moa egg is estimated to have been approximately 80 times the volume of a chicken egg and entirely beyond the carrying capacity of any living avian surrogate, meaning that an artificial incubation system is a prerequisite for the reproductive pipeline rather than an optional supplement. A significant step toward resolving this was taken in May 2026 when Colossal successfully hatched 26 live chicks from a fully artificial incubation platform—a 3D-printed lattice shell incorporating a bioengineered silicone-based membrane that replicates natural eggshell oxygen transfer under normal atmospheric conditions, without supplemental oxygen (Colossal Biosciences, press release, May 2026). The platform is designed to be scaled to moa egg dimensions and represents the first demonstration that complete avian embryonic development outside a biological eggshell is achievable—though the path from chicken proof-of-concept to a genetically edited moa embryo developing to term remains formidable.

Across all programmes, significant limitations extend well beyond the cellular and reproductive pipeline. The ecological implications of introducing proxy organisms into modern ecosystems deserve particular scrutiny. The ecosystems into which de-extinction candidates would be released have changed profoundly since these species last occupied them—habitats have contracted, prey and competitor species have shifted, and novel anthropogenic pressures including disease, climate change, and land use transformation have fundamentally altered the ecological context. A hybrid proxy mammoth released into northern boreal ecosystems would encounter a landscape shaped by two thousand years of ecological succession without megafauna, and the cascading effects of restoring a large browsing herbivore—on vegetation structure, soil carbon dynamics, and the populations of existing species—are genuinely unpredictable [112,113]. The risk is not merely that proxy organisms would fail to occupy their intended niche, but that they could disrupt well-established ecological relationships in ways that create new imbalances rather than restoring old ones. Attempting to resurrect a past ecological baseline in a present ecological context is not ecologically neutral: it is a novel intervention with novel consequences that require independent ecological risk assessment before any release. The dire wolf case makes this concrete—three grey wolves with altered morphology introduced into landscapes where grey wolf populations, prey dynamics, and territorial boundaries are already established pose questions about competitive interactions and hybridisation that have not been addressed at the programme level. Governance frameworks for bioengineered organisms remain absent or ill-suited to these novel entities in most jurisdictions, raising unresolved questions about legal status, liability, containment, and the ecological risk assessment standards that should apply before any reintroduction is attempted.

It bears stating explicitly that no iPSC line from any NMS has yet been used to produce a live birth through any route—not through iSCNT, not through embryo transfer, and not through IVG. The cellular achievements described here—iPSC derivation, multiplex editing, and PGCLC induction—are necessary but far from sufficient conditions for the reproductive outcomes that de-extinction programmes ultimately require. The translational pipeline from an edited iPSC line to a live animal with confirmed in vivo viability and reproductive competence remains undemonstrated in any non-rodent species.

## 4. iPSC Applications in Veterinary and Comparative Medicine

This section examines iPSC applications across companion animal, livestock, and non-human primate systems—collectively framed within the one medicine paradigm, which recognises that animal and human health are mechanistically and epidemiologically interdependent. A defining feature of this research is its bidirectionality: human iPSC methodology accelerates veterinary platform development, while spontaneous animal diseases—arising in outbred, environmentally relevant contexts with authentic genetic complexity—generate mechanistic insights that feed back into human medicine. Four domains are reviewed: regenerative medicine in companion and livestock species, comparative disease modelling, infectious disease platforms for host-restricted pathogens, and zoonotic surveillance. The section closes with xenotransplantation—the application in which porcine iPSC platforms translate most directly into human clinical medicine—as the clearest expression of what the one medicine framework can deliver in practice. The human iPSC clinical translation baseline established in Section 2.3.7—including the first regulatory approvals of iPSC-derived therapies in Japan and ongoing Phase I/II trials in the United States—sets the translational benchmark against which all applications reviewed here will ultimately be measured.

### 4.1. Regenerative Medicine in Companion and Livestock Species

iPSCs offer a compelling alternative to lineage-restricted mesenchymal stem cells for tissue repair, combining unlimited self-renewal with broader differentiation potential [1]. Regenerative applications have advanced furthest in equine, canine, and feline systems, though the gap between cellular proof-of-concept and clinical translation remains substantial across all three.

In horses—where orthopaedic injury is a major clinical and economic burden—equine iPSCs (E-iPSCs) derived from adipose stem cells promote muscle regeneration and form donor-derived myofibres when transplanted into injured mice [114]. Tenogenic differentiation has proven harder: E-iPSCs form inert aggregates rather than functional tendon unless rescued by cyclic mechanical loading or overexpression of the Mohawk transcription factor, and their derivatives are acutely sensitive to the IL-1β and TNF-α that dominate injured tendon, which induces apoptosis and threatens graft survival [115,116]. E-iPSCs therefore remain a research tool, with bone-marrow- and adipose-derived MSCs still the evidence-based standard for equine tendon repair translation [117].

Canine iPSC (ciPSC) work has concentrated on cardiac disease and translational infrastructure. Dogs spontaneously develop dilated cardiomyopathy and degenerative valve disease resembling human heart failure, and canine iPSCs have been differentiated into beating cardiomyocytes expressing cardiac troponin T and MYH6, most recently via a defined AR culture medium (ciPSC medium supplemented with activin and IWR1) yielding spontaneously contracting cells [118,119]. Canine models have also been pivotal to retinal gene therapy—including the preclinical evidence base for Luxturna^®^, the first FDA-approved gene therapy for inherited retinal dystrophy—and that established infrastructure is now being leveraged to adapt human iPSC-derived retinal pigment epithelium protocols for canine inherited retinal degenerations [120,121]. In cats, non-integrative Sendai-virus reprogramming has produced footprint-free feline iPSCs (fiPSCs) from somatic tissues, from which mesenchymal stromal cells and renal cell models have been generated for regenerative and therapeutic-screening applications [45,122].

Across all species, the primary bottlenecks are shared: allogeneic off-the-shelf products risk immune rejection while MHC matching is impractical given the breadth of veterinary MHC diversity; GMP-grade autologous lines are economically prohibitive at scale; and differentiation fidelity together with jurisdictionally fragmented regulatory frameworks further slows translation [123,124]. The regenerative medicine landscape for companion and livestock iPSCs is one of genuine but unevenly distributed progress—with canine cardiomyocyte differentiation, and feline stromal cell alternatives, advancing on the strength of well-characterised disease models and clear human medicine analogues, while equine tendon repair and broadly applicable allogeneic cell therapy remain constrained by differentiation fidelity and inflammatory microenvironment sensitivity. iPSC technology does not yet deliver finished therapeutic products in these species, but it is progressively building the cellular infrastructure—renewable, genetically defined, and editable at the stem cell stage—from which those products will eventually emerge.

### 4.2. Disease Modelling and Comparative Medicine

Companion-animal cardiology illustrates the bidirectionality principle most clearly. Canine dilated cardiomyopathy shares causal genetics with the human disease—dystrophin (*DMD*) and *RBM20* variants drive the condition in specific breeds—so ciPSC-derived cardiomyocytes from affected dogs offer a genetically authentic large-animal platform for mechanistic study and therapeutic testing unavailable in rodents [116].

The feline HCM-causing MYBPC3/R820W mutation was introduced into human iPSC-derived cardiomyocytes to recapitulate hypertrophic cardiomyopathy at the cellular level, demonstrating the translational value of iPSC platforms for studying species-specific cardiac disease [12].

The same logic extends across disease areas. Feline immunodeficiency virus is a naturally occurring lentivirus that mirrors HIV/AIDS in receptor-mediated entry, CD4^+^ T-cell depletion, and neuroinvasion; fiPSC-derived immune-cell populations and brain organoids would enable study of retroviral entry, latency, and neurodegeneration in a species-authentic system [125]. Spontaneous canine tumours share orthologous driver mutations and heterogeneous microenvironments that laboratory-induced rodent models fail to reproduce, making ciPSCs from dogs with naturally occurring cancers a distinctive oncology platform [126]. In livestock, bovine iPSCs carrying the naturally occurring *NPC1* variant found in Angus cattle offer a large-animal model of Niemann-Pick type C disease that translates directly to the rare human paediatric condition [127]. Non-human primate platforms extend the logic to neurodegeneration: marmosets develop age-related Aβ and tau pathology and share human-like brain network organisation, and marmoset iPSC-derived neural organoids carrying *PSEN1* mutations model Alzheimer’s disease under genomic conditions rodents cannot approximate [128,129]. What unites these examples is not merely methodological convergence but a shared translational outcome: in each case, the iPSC-derived model from the affected species has either resolved a mechanistic question that rodent surrogates could not, or opened a therapeutic or vaccine development pathway that was previously inaccessible. The one medicine framework is not simply a conceptual aspiration here—it is being operationalised, disease by disease, in cell types that actually determine pathogen and disease outcome.

### 4.3. Infectious Disease

iPSC-derived cell platforms have proven particularly transformative for studying pathogens with extreme host and cell-type specificity—diseases for which primary cell access is logistically prohibitive, genetically intractable, or ethically constrained [130,131]. The unifying principle is that iPSC derivation converts a scarce, terminally differentiated, manipulation-resistant target cell type into a renewable, genetically defined, CRISPR-amenable platform—and this conversion is especially consequential for pathogens whose host range is narrow and whose biology cannot be adequately studied in heterologous systems.

The most extensively developed example is African Swine Fever Virus (ASFV), which replicates almost exclusively in porcine macrophages—terminally differentiated, difficult to culture in quantity, and resistant to stable genetic manipulation. iPSC-derived macrophages that support productive ASFV and PRRSV infection and are amenable to CRISPR editing at the stem cell stage circumvent this bottleneck entirely [11]. A genome-wide knockout screen in porcine iPSC-derived macrophages identified the non-classical MHC II molecule SLA-DM as an essential host factor for ASFV replication, with SLA-DMA and SLA-DMB knockout producing severe replication defects confirmed by transgenic reconstitution—establishing SLA-DM editing in porcine iPSCs as a candidate route to ASF-resistant animals [132]. That CD163 knockout confers no ASFV resistance, despite conferring complete PRRSV resistance, confirms that these viruses require distinct host factors and validates SLA-DM as an independently identified iPSC editing target [133]. Extending the ASFV platform across Suidae—domestic pig, wild boar, and warthog—has further enabled direct in vitro recapitulation of the species-differential tolerance phenotype observed in vivo: warthog iPSC-derived macrophages fail to induce interferon-α upon ASFV challenge, a finding directly relevant to vaccine design and spillover risk assessment [51].

Feline infectious peritonitis virus (FIPV) presents an analogous macrophage-tropism problem in companion animal medicine. FIPV arises through mutation of feline enteric coronavirus and causes a near-universally fatal systemic disease characterised by monocyte and macrophage infection, vasculitis, and immune-mediated tissue destruction [134,135]. Antibody-dependent enhancement further complicates vaccine development by facilitating viral uptake by macrophages via non-neutralizing antibodies, making mechanistic dissection of macrophage–virus interactions a priority [136,137]. fiPSC-derived macrophages and monocytes would provide the first genetically manipulable, renewable platform for studying FIPV entry, latency, ADE mechanisms, and antiviral candidate screening in species-authentic cells—a direct analogue to the ASFV macrophage system, and one for which the fiPSC derivation infrastructure now exists [45].

Bat iPSC platforms have opened a promising but still nascent angle on infectious disease: interrogating why bats harbour viruses that are highly pathogenic to humans and other animals without themselves succumbing to disease. Bat iPSCs were first derived from *Myotis lucifugus* using a piggyBac vector delivering eight reprogramming factors, establishing that chiropteran somatic cells are amenable to reprogramming despite their divergent biology [138]. Subsequent work derived iPSCs from *Rhinolophus ferrumequinum*—a horseshoe bat species closely related to the likely SARS-CoV-2 reservoir—and revealed large virus-filled vesicles persisting in pluripotent cells without compromising proliferation, suggesting an unusual entanglement between bat stem cells and endogenous viral elements. A further study compared SARS-CoV-2 infection of human and bat iPSCs following ACE2 transduction, finding substantially lower infectious virus titres and cytotoxicity in bat iPSCs, with bat embryonic fibroblasts showing fully abortive infection despite productive viral RNA and protein synthesis [139]. These findings are intriguing, but their translational value is currently limited by a critical gap: bat iPSCs have not yet been successfully differentiated into the tissue-specific cell types—respiratory epithelium, macrophages, hepatocytes—that would be needed to model infection at ecologically and clinically relevant target sites. In this respect, bat organoid systems derived directly from adult tissue have thus far proven more tractable: horseshoe bat intestinal organoids support robust SARS-CoV-2 replication [140], and more recently, bat nasal, bronchial, alveolar, and small intestinal organoids have been used to model Marburg virus infection and characterise species-specific antiviral responses at mucosal surfaces [141]. The iPSC route retains its distinctive advantages—genetic manipulability at the stem cell stage, indefinite expandability, and applicability to species from which adult tissue cannot be obtained—but realising these advantages for bat infectious disease biology requires the development of directed differentiation protocols into relevant epithelial and immune lineages, which remains an unmet priority for the field.

Across all three examples—porcine macrophage-tropic viruses, feline coronavirus, and bat reservoir biology—the iPSC framework enables a mode of investigation that is simply not achievable through conventional primary cell or heterologous model approaches: genetically defined, species-authentic, renewable infection modelling in the cell types that actually determine pathogen outcome. The cross-species comparative approach illustrated by the Suidae ASFV platform—in which host-restricted pathogen biology is interrogated simultaneously across phylogenetically diverse species—points directly toward the broader One Health application addressed in the following subsection.

### 4.4. iPSC-Based Models for Zoonotic Disease and One Health

The WHO One Health framework, most recently formalised in the May 2025 Pandemic Agreement, codifies as a priority integrated surveillance at the human–animal–environment interface—a priority that iPSC technology is conceptually well-positioned to support (WHO 2025). By generating species-specific iPSC-derived organoids from reservoir hosts, intermediate hosts, and humans, it is in principle possible to model discrete stages of viral spillover—reservoir tolerance, intermediate host adaptation, and human susceptibility—within a controlled, reproducible system without the ethical and logistical constraints of multi-species in vivo infection studies [142,143,144]. Multi-species respiratory organoid panels, for instance, could enable rapid assessment of emerging pathogen tropism and adaptation potential at the earliest stages of a spillover event.

This case is compelling in concept but must be stated honestly: no validated multi-species iPSC spillover model currently exists, and the pathway from proof-of-principle organoid infection studies to actionable surveillance tools remains undefined. The value of retaining this framework in the present review is prospective—to position NMS iPSC platforms as infrastructure with One Health utility before that utility is formally demanded—rather than to describe an established application.

### 4.5. Xenotransplantation

Porcine iPSCs (piPSCs) hold particular promise as a starting material for xenotransplantation, given the anatomical and physiological proximity of porcine and human organs. The iPSC route is advantageous because humanisation edits—introducing complement regulatory genes and knocking out xenoantigens—can be performed and validated at the stem cell stage before differentiation and organogenesis, rather than requiring a whole transgenic animal for each modification [145,146]. Clinical xenotransplantation from iPSC-derived sources remains a long-term prospect, but pig-to-human organ transplants using genetically modified animals are already entering early clinical evaluation, establishing the translational trajectory into which iPSC-derived porcine sources will eventually feed [147,148]. The same principle that makes porcine iPSCs valuable for xenotransplantation—that editing and quality control performed at the stem cell stage before any commitment to whole-animal production offers precision and scalability unavailable through direct embryo or primary cell approaches—applies with equal force to the disease modelling and infectious disease platforms described in the sections that follow.

## 5. Agriculture and Biotechnology

iPSC are increasingly being applied across livestock species to address core challenges in modern agriculture: improving productivity and disease resistance, preserving genetic diversity in the face of climate change, and enabling novel biotechnological applications including cultured meat. Across pigs, cattle, and chickens, iPSC platforms are beginning to move beyond proof-of-concept toward applied tools for sustainable and resilient food systems [149].

Porcine iPSCs (piPSCs) were first derived in 2009 from foetal and postnatal fibroblasts using OSKM factors [150] and have since been refined for targeted genetic modification aimed at both productivity enhancement and disease resistance [151,152,153,154]. Bovine iPSCs (biPSCs) offer a platform not only for genetic trait improvement but also for developing stem cell-based therapies for inherited disorders such as citrullinemia and leukocyte adhesion deficiency in cattle [155,156]. More broadly, ruminant iPSCs—spanning cattle, sheep, goats, and buffalo—support genetic improvement programs, disease resistance breeding, and biomanufacturing applications including cultured meat and dairy [149]. Avian iPSCs represent an emerging frontier: the chicken’s short life cycle and well-characterised genome make it particularly tractable for stem cell studies, and chicken iPSCs are being applied to genetic engineering, production trait improvement, and germline transmission to fix desirable variants in breeding populations [157,158].

### 5.1. Disease Resistance and Modelling

iPSC platforms enable two complementary approaches to agricultural disease challenges: resistance breeding through precision genome editing, and in vitro disease modelling for therapeutic and vaccine development. The mechanistic basis of these platforms—particularly the use of iPSC-derived macrophages for host-restricted pathogen biology—is developed in detail in Section 4.3; the focus here is the resistance breeding logic and its agricultural implications.

The core argument is straightforward: editing immune-related loci in iPSCs, followed by SCNT or embryo production from edited lines, offers a route to generating livestock with heritable, precisely defined disease resistance without the stochasticity of conventional breeding. The iPSC route carries a critical advantage over direct embryo editing in this context: edits can be introduced, screened, and quality-controlled at the stem cell stage across large cell numbers before any commitment to embryo production, enabling multiplex strategies and rigorous off-target assessment that are impractical at the zygote level.

The strongest proof-of-concept comes from the CD163/PRRSV system, in which deletion of scavenger receptor cysteine-rich domain 5 confers complete and heritable PRRSV resistance in pigs, with FDA approval granted in April 2025 for commercial use [159,160]. While achieved through direct embryo editing rather than an iPSC pipeline, it validates the principle that editing a single immune-related locus can produce commercially viable, heritable disease resistance at scale. For ASFV—which has no licensed vaccine and has killed more than 200 million pigs worldwide—the SLA-DM locus identified through iPSC-derived macrophage screens (Section 4.3) represents the leading iPSC-specific editing target, with translation toward live animal models underway at the Roslin Institute [132]. This remains a cellular-level finding; in vivo confirmation in edited animals is the critical prospective step. The amenability of chicken iPSCs to targeted genome editing opens analogous resistance engineering opportunities in poultry—including potential targets relevant to highly pathogenic avian influenza, the most economically and epidemiologically consequential poultry disease globally -complementing conventional selective breeding approaches in avian production species [157,161].

### 5.2. Climate Resilience and Genetic Diversity Preservation

Climate change poses an escalating threat to livestock productivity, particularly through heat stress, shifting disease landscapes, and the erosion of locally adapted breeds. iPSC technology offers two distinct strategies to address this.

The first is active genetic intervention. The “slick” gene variant in cattle, which produces a short coat and measurably reduces heat stress, illustrates how iPSC-based editing can accelerate the introgression of climate-adaptive traits: The ‘slick’ gene variant in cattle, which produces a short coat and measurably reduces heat stress, illustrates the potential of iPSC-based editing for accelerating climate-adaptive trait introgression: the variant can be introduced by gene editing in bovine iPSCs at the stem cell stage—enabling rigorous quality control before embryo commitment—though the full pipeline from iPSC-edited cells to live heat-tolerant calves via SCNT or embryo transfer has not yet been demonstrated and represents a prospective application of the iPSC route [162]. The recent generation of stable bovine induced totipotent stem cells (iTPSCs) has further expanded this toolkit [163]. Unlike conventional iPSCs, which are restricted to embryonic lineages, iTPSCs retain the capacity to contribute to both embryonic and extraembryonic tissues—a property that enables more efficient whole-animal cloning from edited cell lines and broadens the reproductive deployment options for climate-adaptive variants. Derived using specific signalling inhibitors alongside the SV40 large T antigen, bovine iTPSCs provide an inexhaustible, cryopreservable cellular substrate for climate-adapted breeding programmes that is not constrained by gamete availability or collection logistics.

The second strategy is preservation. A living iPSC biobank—in which somatic cells from locally adapted breeds are reprogrammed and banked as iPSCs rather than as terminally differentiated cells—creates a living, expandable genetic resource that may in future be differentiated into germline-competent cells and reintroduced into breeding programs, though germline competence from bovine iPSCs has not yet been demonstrated and remains a key unresolved step before this strategy becomes operationally applicable [65]. Unlike conventional cryobanking of sperm or embryos, iPSC banks are not limited by collection logistics or gamete availability, and the cells remain amenable to future editing as new climate-adaptive targets are identified.

### 5.3. Cultured Meat

iPSCs are increasingly positioned as a foundational cell source for cultured meat production, offering an indefinitely self-renewing starting material that can be differentiated into skeletal muscle and adipose tissue at scale [164]. Species-specific iPSC lines from cattle [165] and pigs [166] have been expanded in serum-free suspension cultures—a prerequisite for cost-effective biomanufacturing—and companies including MyriaMeat and Magic Valley are already leveraging iPSC-derived platforms to produce cultivated meat products [167].The projected sustainability case for iPSC-derived meat is substantial: production from a single biopsy eliminates slaughter and reduces antibiotic use, and lifecycle analyses project significantly lower land, water, and greenhouse gas footprints than conventional livestock farming at commercial scale [164]. These projections are, however, modelling-dependent and contingent on resolving current production inefficiencies—particularly the energy-intensive bioreactor conditions and costly growth media formulations that currently dominate the environmental footprint of cultivated meat production—meaning that the realized environmental benefit at scale remains to be demonstrated empirically. For wildlife conservation, species-specific iPSC lines from animals that cannot be farmed conventionally, including bushmeat species under poaching pressure, could provide culturally relevant protein alternatives without any wild harvest—though these applications remain speculative.

Large-scale viability depends on overcoming persistent challenges: the cost of growth media remains the dominant production bottleneck; scaffold technology for replicating whole-cut muscle texture rather than ground products remains an unresolved bioengineering challenge; and consumer acceptance is complicated by naturalness concerns and labelling ambiguity across major markets [168].

The regulatory landscape is fragmented and rapidly evolving. Singapore approved a cultivated meat product in 2020, followed by joint FDA/USDA clearance for GOOD Meat and Upside Foods in 2023, while the European Union is reviewing applications with no approvals to date, and Italy has legislated an outright ban [168]. For iPSC-specific products, an additional regulatory layer applies: no jurisdiction has issued specific guidance on iPSC-derived food ingredients, and open questions regarding residual pluripotency, genomic stability of extensively passaged lines, and the acceptability of reprogramming vectors in a food production context will need to be resolved before iPSC-derived cultured meat can advance through approval pipelines. Engaging regulators early to define the required evidence standards will be as important as solving the remaining biological challenges.

## 6. Environmental Protection

Conventional ecotoxicological assessment has relied on a narrow repertoire of model organisms—zebrafish embryos, *Daphnia*, rat hepatocyte lines, and standardised Organisation for Economic Co-operation and Development (OECD) bioassays—that inadequately represent the physiological and metabolic diversity of wild species [169,170]. Extrapolating toxicity data from these models to ecologically relevant wildlife involves assumptions about receptor conservation, metabolic equivalence, and developmental timing that are frequently violated across the phylogenetic distances involved. Non-human iPSC-derived cells offer a route to interrogate contaminant effects in a species-, tissue-, and developmental-stage-specific manner, with the capacity to resolve mechanisms that whole-animal tests obscure and to generate data directly relevant to species of conservation concern.

Four toxicological mechanisms are particularly well-suited to iPSC-based investigation in wildlife contexts: AHR-mediated toxicity (dioxins, PCBs, PAHs) [171], endocrine disruption (EDCs via nuclear receptor signaling) [172], oxidative stress and heavy metal toxicity (NRF2/KEAP1 pathway) [173], and developmental toxicology (WNT/BMP/NODAL signalling, gastruloid models) [174]. For transparency, the demonstrated versus aspirational status of each application is stated explicitly throughout this section and summarized here. What has been demonstrated in NMS: iPSC derivation and cryopreservation from mammalian wildlife species, enabling in principle the establishment of renewable cellular platforms for ecotoxicological assays [9]; NRF2/KEAP1 pathway characterization in human and rodent iPSC-derived cells, providing the methodological foundation for wildlife extension [173]; and frog primary fibroblast cultures applied to ecotoxicological assays, representing limited but genuine progress in amphibian systems [175]. What has not yet been demonstrated in any NMS: AHR toxicology assays using iPSC-derived hepatocyte-like or gill-like cells; EDC assays using iPSC-derived steroidogenic or thyroid lineages; gastruloid-based developmental toxicology models; NRF2/KEAP1 characterization in any wildlife iPSC platform; and stable iPSC derivation in fish or amphibian species [176]. The mechanistic rationale for all four application areas is well-founded; the technical infrastructure to realise them is not yet in place for any NMS. This assessment is based on a comprehensive search of PubMed, Web of Science, and Scopus using terms combining iPSC or induced pluripotent stem cell with wildlife, non-model species, ecotoxicology, AHR, endocrine disruption, NRF2, oxidative stress, and developmental toxicology; to our knowledge, no published study has reported any NMS iPSC-derived ecotoxicological assay for any of the four mechanisms reviewed here [170].

### 6.1. iPSC-Derived Cell Systems as Species-Authentic Reporters of Organic Pollutants

Dioxins, PCBs, and polycyclic aromatic hydrocarbons represent among the most ecologically consequential persistent organic pollutants in aquatic and terrestrial environments, and their primary mechanism of cellular toxicity—activation of the aryl hydrocarbon receptor (AHR)—is one of the least adequately captured by current standard assay species. The AHR axis operates via bHLH/PAS-domain-dependent nuclear translocation, ARNT dimerisation, and transcriptional induction of CYP1A1 and CYP1B1, with downstream consequences that extend beyond xenobiotic metabolism to include mitochondrial dysfunction and G1/S cell-cycle arrest via direct interaction with hypophosphorylated retinoblastoma protein—effects with direct implications for tissue renewal and reproductive output [177,178,179,180]. The central problem for ecological risk assessment is that AHR ligand affinity varies up to 1000-fold across vertebrate species, meaning that a contaminant concentration tolerated by a rat hepatocyte may be acutely toxic to a sentinel fish, raptor, or marine mammal—divergences that human or rodent-based regulatory assays are structurally unable to detect [181]. No NMS iPSC-derived cell system has yet been applied to this problem, despite the fact that the scientific rationale is among the strongest of any mechanism reviewed here. iPSC-derived hepatocyte-like or gill-like cells from species with characterised AHR polymorphisms would enable population-specific mechanistic interrogation at precisely the level of biological resolution that risk assessment requires. Atlantic killifish populations independently adapted to Superfund-site PCB contamination via convergent AHR pathway desensitisation represent a particularly compelling proof-of-concept target: the genetic basis of tolerance is characterised, the ecological stakes are high, and the contrast between sensitive and resistant populations within a single species offers a controlled system for dissecting AHR-mediated toxicity without cross-species confounding [182,183].

### 6.2. Organic Pollutants and Endocrine Disruption

As with AHR toxicology, no NMS iPSC-derived steroidogenic or thyroid lineage has been deployed in an Endocrine-Disrupting Chemicals (EDCs) assay context. The gap this creates is significant. EDCs perturb nuclear receptor signalling through ERα, AR, and TR pathways [184], as well as non-nuclear receptor mechanisms including AHR-ERα cross-talk, in which ligand-bound AHR directly suppresses oestrogenic gene expression [185]. Most validated in vitro EDC assays use rodent or human receptor constructs that may substantially misrepresent potency in wildlife species with divergent ligand-binding domains [186]. Species-specific iPSC-derived steroidogenic or thyroid lineages could directly address this limitation [187], though validated differentiation protocols for any wildlife species do not yet exist and represent a significant unmet technical need.

### 6.3. iPSC-Derived Platforms for Oxidative Stress and Heavy Metal Toxicity

ROS-mediated oxidative stress induced by heavy metals, nanomaterials, and pesticides triggers NRF2/KEAP1 stress responses, mitochondrial dysfunction, and apoptosis [188,189,190]. Here, the potential contribution of NMS iPSC platforms is practical as much as mechanistic. Primary cells from rare or protected species are difficult to obtain in quantities sufficient for replicated dose–response experiments, and are legally prohibited to collect repeatedly in most jurisdictions. iPSC-derived cells would provide a renewable, genetically defined alternative that overcomes both the supply constraint and the inter-preparation variability inherent to primary cell isolation [191,192]. While NRF2 pathway characterisation using iPSC-derived cells has been demonstrated in human and rodent systems [193], its application in wildlife iPSC platforms remains largely unrealised; establishing baseline NRF2/KEAP1 signalling profiles in iPSC-derived cells from species with documented heavy metal exposure histories—cetaceans, raptors, and freshwater fish from contaminated catchments—represents a tractable near-term priority [194,195,196].

### 6.4. iPSC-Based Gastruloid Models for Developmental Toxicology

iPSC-based embryoid body and gastruloid models have been used to assess teratogenicity via perturbation of WNT, BMP, and NODAL/ACTIVIN signalling axes in human and rodent systems, providing an alternative to whole-embryo tests that is both more mechanistically informative and more ethically tractable for protected species [197,198]. Extension to NMS has not been attempted. Doing so requires species-specific baseline characterisation before such models can be deployed with confidence—specifically, confirmation that WNT/BMP signalling dynamics during gastruloid formation mirror in vivo developmental gene expression in the target species, which in turn requires single-cell transcriptomic reference data from early embryos that exist for very few NMS [199,200,201,202]. As these genomic resources improve [203,204], developmental iPSC models will become increasingly deployable across ecologically relevant taxa—but this remains a second-order priority contingent on first establishing stable, validated iPSC lines and basic differentiation capacity in the species of interest.

### 6.5. From Aspiration to Action: Prioritising NMS iPSC Investment Across Toxicological Mechanisms

Across these four mechanisms, a common thread is that the mechanistic specificity enabled by iPSC-derived wildlife cells represents a qualitative advance over what conventional ecotoxicological models can provide [170,205]: not merely a more convenient assay format, but access to species-authentic receptor biology, developmental context, and tissue-specific metabolism that are invisible to standardised bioassays [206]. The honest picture, however, is that none of these applications yet exists in practice for NMS. The most tractable near-term entry points are those where the upstream requirements are already partially met: mammalian wildlife iPSC lines that exist today could be directed toward NRF2/KEAP1 characterisation without new reprogramming investment; killifish iPSC derivation, if achieved, would immediately unlock AHR population toxicology; and EDC receptor assays could be built on primate iPSC lines already established for conservation purposes. Progress across all four mechanisms is contingent on the same upstream investments: improved genome annotation for non-model species [207], development of species-specific differentiation protocols [200] and establishment of in vivo transcriptomic reference datasets against which iPSC-derived cell identity can be validated [208,209]. Until these foundations are in place, wildlife iPSC-based ecotoxicology will remain largely aspirational—but the scientific case for building them is compelling, and the tools to do so are rapidly maturing.

### 6.6. Taxonomic Readiness for iPSC-Based Ecotoxicology: Mammals, Fish, and Amphibians

The practical utility of iPSC-based ecotoxicology depends entirely on the availability of stable, validated cell lines, and this varies enormously across taxonomic groups. In mammalian wildlife, robust iPSC derivation and partial differentiation have been achieved across a growing number of species, and the prospect of “living biobanks” that integrate toxicological screening capacity with genetic preservation is realistic [8]. Here, iPSC platforms offer the most immediately actionable value, enabling chronic, sub-lethal, multi-compound exposure studies in species of direct conservation concern without requiring repeated tissue collection from protected animals.

Fish-derived iPSC platforms would substantially extend ecotoxicological capabilities to aquatic species of conservation relevance, enabling tissue-type-specific interrogation of contaminant exposures that accumulate across developmental time [210]. However, whether this is achievable in the near term depends critically on establishing stable, well-validated fish iPSC lines—a goal not yet demonstrably met for any species. Recent progress in chemical reprogramming of koi caudal fin fibroblasts using a staged chemically defined medium represents meaningful but preliminary progress; pluripotency validation and directed differentiation into ecotoxicologically relevant cell types remain to be demonstrated [211].

The case for amphibian iPSC development is arguably the most urgent. The permeable skin, biphasic life history, and exquisite sensitivity of amphibians to contaminants, thermal stress, and pathogens such as *Batrachochytrium dendrobatidis* make them ideal cellular sentinels for environmental monitoring. Recent frog primary fibroblast cultures applied to ecotoxicological assays [175] represent genuine but limited progress—these are not renewable iPSC platforms, and the fundamental reprogramming milestones remain unachieved for any amphibian species. This is the largest gap between ecological urgency and technical readiness in the entire ecotoxicology section and closing it should be treated as a priority for the field.

Scaling iPSC-based environmental monitoring across the hundreds of threatened species for which such platforms would be most valuable faces formidable logistical challenges: each species requires bespoke reprogramming optimisation, pluripotency validation, and differentiation protocol development. Prioritisation frameworks—identifying sentinel species that are both ecologically informative and technically tractable—will be essential if the field is to allocate limited resources effectively.

### 6.7. iPSC-Based Environmental Toxicology in NMS: What Is Demonstrated, What Remains Aspirational

Non-human iPSC technology occupies an intellectually compelling but technically uneven position in environmental science. Its most clearly demonstrated value lies in mammalian wildlife, where robust derivation and practical biobanking are achievable today, and where the path from existing iPSC lines to rudimentary toxicological assays is shorter than in any other taxonomic group. For aquatic and amphibian taxa—arguably the most ecologically urgent targets—iPSC platforms remain aspirational, constrained by unresolved reprogramming challenges and the absence of validated pluripotency standards. The mechanistic arguments for deploying these systems in AHR toxicology, EDC assessment, developmental toxicology, oxidative stress and heavy metal toxicity are well grounded, but none has been realised in practice for NMS, and realising them requires species-specific technical investment not yet commensurate with the ambition. Used critically and with transparency about current limitations, non-human iPSC technology has genuine potential to strengthen environmental protection; used uncritically, it risks overpromising at the expense of the field’s credibility.

## 7. Conclusions

The trajectory of iPSC science across NMS over the past fifteen years is one of genuine, if uneven, progress. From the first proof-of-concept lines derived from a drill and a Northern white rhinoceros in 2011, the field has expanded to encompass megafauna, marsupials, monotremes, primates, birds, bats, and early forays into fish—spanning applications from wildlife genetic rescue and veterinary regenerative medicine to agricultural disease resistance, cultured meat, and ecotoxicological assessment. Taken together, this body of work makes one conclusion difficult to avoid: the deep conservation of the core pluripotency network across vertebrates suggests that reprogramming may, in principle, be achievable across a far broader range of species than currently demonstrated. What primarily limits the field is infrastructure—the absence of annotated reference genomes, validated species-specific reagents, standardized culture conditions, agreed quality control frameworks, and the institutional funding mechanisms that would make systematic protocol development feasible at scale—though species-specific biological barriers, including pluripotency state diversity, divergent signaling dependencies, and reagent incompatibility, compound this deficit and must be resolved alongside it.

This infrastructure deficit manifests at every stage of the iPSC workflow. Pluripotency state diversity across vertebrates remains poorly characterized: the naïve, formative, and primed states defined in mouse and human have no confirmed equivalents in most NMS, and without single-cell embryogenesis atlases to define the in vivo target, reprogramming outcomes in novel species cannot be benchmarked against a biological ground truth. Culture incompatibility compounds this uncertainty—commercially available maintenance media optimized for human or murine cells routinely fail in NMS, while the species-specific growth factor requirements that would guide rational medium design are almost entirely unknown. Factor choice presents a parallel challenge: canonical OSKM combinations frequently underperform in species whose pluripotency networks have diverged from the mammalian blueprint, and the identification of optimal species-matched or species-supplemented factor sets requires genomic and transcriptomic resources that most NMS lack. The shift toward non-integrative delivery—Sendai virus, episomal vectors, and chemically defined reprogramming—has meaningfully reduced the genomic footprint of NMS iPSC lines and brought conservation and reproductive applications closer to practical feasibility. But non-integrative efficiency remains lower than viral methods, and optimizations across taxonomically diverse cell types is far from complete.

An equally practical but underappreciated operational constraint is the choice of starting cell source. For most wildlife species, the default approach—skin biopsy for fibroblast isolation—is invasive, requires physical capture or sedation, and may be ethically restricted or logistically impossible for free-ranging, critically endangered, or legally protected animals. Non-invasive alternatives exist and have been successfully applied in NMS: peripheral blood mononuclear cells (PBMCs), obtainable from small blood draws during routine health assessments have been used to generate iPSCs from porcine [72,73]; urine-derived cells—renal epithelial cells shed in voided urine—have been reprogrammed into iPSCs in human and primate systems and represent a genuinely non-invasive option applicable to species for which even blood sampling is constrained [212,213]; and pinfeather pulp cells from developing feathers have enabled iPSC derivation in avian species without the need for surgical intervention [60]. Exfoliated cells from nasal or oral swabs and faecal-derived cells are emerging alternatives under investigation for additional taxa. Even where a minimally invasive cell source is secured, the capacity of those cells to survive primary culture, attach, proliferate, and withstand the stress of reprogramming is itself species- and tissue-dependent—a bottleneck that receives less systematic attention in the literature than factor choice or delivery method, but that determines whether a reprogramming attempt is even possible. Strategic investment in non-invasive collection protocols and in understanding the survival and proliferation characteristics of primary cells from diverse wildlife species represents an underappreciated but operationally critical frontier for the field.

Functional validation represents perhaps the deepest unresolved challenge. In the absence of germline-competent chimera formation—a benchmark achieved for NMS in only a handful of cases—the developmental potency of most published NMS iPSC lines remains formally unverified. Surrogate validation criteria such as pluripotency marker expression, embryoid body formation, and teratoma assays, while informative, are insufficient to exclude the possibility that cells represent partially reprogrammed intermediates. The field would benefit substantially from agreed minimum reporting standards that specify which combination of molecular, functional, and genomic quality control steps constitutes an acceptable evidence base for a published NMS iPSC line-standards calibrated to be scientifically rigorous while remaining practically achievable given the logistical realities of working with protected or rare species. A minimum evidence base is proposed in Section 2.3.6 and comprises five elements: pluripotency marker expression confirmed for species-appropriate orthologues at transcript and protein level; transgene silencing confirmation by exogenous-specific RT-PCR; functional pluripotency demonstrated by tri-lineage differentiation or teratoma assay; genomic integrity confirmed by karyotyping at a defined early passage; and stable self-renewal for a minimum of ten passages. Lines meeting all five criteria may be described as bona fide iPSCs; lines meeting fewer should be described as putative iPSC-like cells or partially reprogrammed intermediates.

The applications reviewed here are not equally mature. In mammalian wildlife conservation, iPSC derivation and early downstream applications—PGCLCs induction, organoid generation, naïve pluripotency capture—are demonstrably achievable today, and the case for building living cellular biobanks as a genetic safety net for critically endangered species is scientifically and practically compelling. In veterinary medicine, iPSC-based disease modelling for companion animal conditions is advancing toward mechanistic utility, and the one medicine framework positions these platforms as genuinely bidirectional contributors to both veterinary and human biomedical knowledge. In agriculture, the CD163/PRRSV and SLA-DM/ASFV proof-of-concept findings demonstrate that iPSC-mediated genome editing can identify and validate disease-resistance targets at cellular resolution before any commitment to animal production—a workflow advantage over embryo-level editing that will only grow as multiplex editing demands increase. In ecotoxicology and comparative medicine, the scientific rationale for species-authentic iPSC-derived cell systems is well-grounded, but the platforms themselves are largely aspirational for non-mammalian taxa, constrained by the same upstream deficits in reprogramming optimisation and genomic annotation.

Looking forward, four investments are most likely to determine the pace at which iPSC technology fulfils its potential across NMS. First, expanded genomic and transcriptomic infrastructure—high-quality reference genome assemblies, single-cell preimplantation atlases, and comparative pluripotency gene regulatory network maps—will simultaneously accelerate factor design, culture optimisation, and reprogramming fidelity assessment across taxonomic groups. Initiatives such as the Earth BioGenome Project are directly enabling this, and the iPSC field should ensure it is positioned to translate genomic outputs into reprogramming reagents and validated protocols as reference data become available. Second, the development of species-specific differentiation protocols—particularly for germline, hepatic, neural, and immune lineages—will determine whether iPSC lines remain cellular curiosities or become functional platforms for conservation, disease modelling, and therapeutic development. Third, governance frameworks suited to the novel biological entities produced by NMS iPSC research—blastoids, genome-edited surrogates, iPSC-derived gametes from functionally extinct species—must be developed in parallel with the science rather than after it. The current absence of regulatory guidance for these entities in most jurisdictions represents a structural vulnerability that could impede translation at precisely the moment when biological feasibility is achieved. Fourth, strategic prioritisation of species for iPSC investment—concentrating effort on taxa that are conservation-critical, technically tractable, and capable of generating broadly transferable methodological insights—will be essential in a field where per-species protocol development remains resource-intensive.

The iPSC revolution that transformed human biomedical research over the past two decades is now, demonstrably, extending beyond its original boundaries. The cells, the concepts, and increasingly the tools are in place. What remains is the systematic, coordinated, and adequately funded effort to build the species-specific knowledge base that will allow those tools to be used with the precision and confidence the stakes demand. For species on the edge of extinction, for companion animals whose diseases mirror our own, for livestock systems under pressure from climate and pathogen alike, and for ecosystems whose sentinel species are being silently compromised by chemical exposures our current assays cannot detect—the case for making that investment is not merely scientific; it is urgent.

## Figures and Tables

**Table 1:** A summary of the five domains, current maturity and main bottlenecks.

Domain	Key Applications	Main Bottlenecks
Section 1 Technical reprogramming	IPSC derivation, non-integrative delivery, species-specific factor combinations, quality contro	Unknown pluripotency ground state in most NMS; absence of single-cell embryogenesis reference data; inconsistent reagent cross-reactivity; no agreed minimum validation standard
Section 2 Conservation and genetic rescue	Biobanking, iSCNT, in vitro gametogenesis, PGCLC derivation, de-extinction	Gonadal somatic niche reconstitution unresolved beyond rodents; spermatogenesis beyond spermatid stage not achieved in NMS; de-extinction programmes cannot achieve true resurrection from reconstructed sequence alone
Section 3 Veterinary & comparative medicine	Regenerative medicine, disease modelling, infectious disease platforms, xenotransplantation, One Health	Allogeneic immune rejection; GMP-grade autologous lines economically prohibitive; bat iPSCs not yet differentiated into relevant infection target cells; no validated multi-species spillover model
Section 4 Agriculture and biotechnology	Disease resistance engineering, climate resilience, cultured meat	SLA-DM editing demonstrated only at cellular level - in vivo confirmation pending; cultured meat cost reduction and regulatory approval unresolved; avian iPSC differentiation protocols underdeveloped
Section 5 Ecotoxicology and environmental protection	AHR toxicology, EDC assessment, oxidative stress, developmental toxicology, sentinel species monitoring	No NMS iPSC ecotoxicology assay yet validated; fish and amphibian iPSC derivation unachieved; species-specific differentiation protocols to hepatocyte-like and steroidogenic lineages absent; OECD guideline integration undefined

Field maturity: Most mature Advancing Early-mid Aspirational.

**Table 2:** Summary of the primary challenges identified across Section 2.2, and the methodological responses developed in Section 2.3.

Challenge	Specific Problems (Section 2.2)	Status in NMS	Solutions and Advances (Section 2.3)
Pluripotency state instability and culture incompatibility	Unknown optimal pluripotency state (naïve, formative, or primed) for most NMS	Prospective	No current solution—near-term priority: single-cell preimplantation atlases; Earth BioGenome Project
	No single-cell embryogenesis reference data for most NMS to define reprogramming targets	Prospective	
	Standard conditions (2i/LIF, FGF/Activin) routinely fail, causing differentiation or growth arrest	Demonstrated	Use of alternative small-molecule cocktails that stabilise pluripotency states
	Unpredictable culture variables: feeder choice, coating substrate, oxygen tension, passaging method, seeding density, small-molecule timing	Partial	Parallel factorial screening of culture conditions from outset rather than sequential optimisation
Genomic and molecular limitations	Incomplete genome assemblies prevent accurate ortholog identification and promoter analysis	Partial	Comparative genomics using existing model species references as scaffolds (Earth BioGenome Project as the near-term systematic solution)
	Validated antibodies against OCT4, SOX2, NANOG, SSEA antigens show inconsistent cross-reactivity in NMS	Partial	Independent validation required; species-specific antibody development or epitope-tagged knock-in lines as longer-term solutions.
	unsilenced reprogramming transgenes	Demonstrated	Non-integrative delivery methods that eliminate transgene silencing as a confound; shRNA-mediated knockdown
	Karyotypic instability during prolonged culture	Partial	Regular karyotyping at defined passage intervals
	Unpredictable transgene silencing efficiency—the apparent self-renewal may reflect ongoing transgene activity	Demonstrated	Examine transgene expression (e.g., exogenous-specific RT-PCR)
Functional validation challenges	Germline-competent chimera formation (gold standard) unachievable for most NMS	Partial	Surrogate criteria accepted in combination as the practical alternative; blastoid generation and organoid formation as additional functional readouts where chimera formation is impractical.
	Surrogate criteria (alkaline phosphatase, marker expression, EB formation, teratoma) cannot exclude partially reprogrammed intermediates	Partial	Agreed minimum evidence base: pluripotency markers + functional assay + genomic QC + transgene silencing confirmation
	No agreed minimum validation standards—published lines vary widely in evidence quality	Prospective	Develop NMS-specific minimum validation standards through field consensus, analogous in process to the ISSCR guidelines
Cross-species reagent incompatibility	Secreted ligand–receptor level: LIF species-specificity; recognition vs. functional response frequently conflated	Demonstrated	Species-specific factor substitution where annotated genomes permit
	Intracellular factor level: avian POU5F3/SOX3/KLF divergence; OSKM engages only partial network; NANOG and LIN28 stalling in birds	Demonstrated	Auxiliary factor supplementation—NANOG, LIN28, KDM4A—to bridge network gaps
	Innate sensing level: species-specific interferon responses reduce Sendai virus efficiency independent of pluripotency signalling	Partial	Engineered super-factors (Super-SOX, MyoD fusion) for enhanced transcriptional potency
			Vector switching (episomal, saRNA) when innate immune barrier—not pluripotency—limits Sendai efficiency
			Pathway-level readouts (pSTAT3, pSMAD2/3, pERK) required before attributing failure to reagent incompatibility

## Data Availability

No new data were created or analyzed in this study. Data sharing is not applicable.

## Supplementary Materials

The following supporting information can be downloaded at: https://www.mdpi.com/article/10.3390/cells15171565/s1, Supplementary Table S1: Timeline of iPSC derivation milestones across non-model species, 2011–2024, with cell source, reprogramming method, factors used, integration status, pluripotency validation assays reported, differentiation demonstrated, and major application domain for each species entry. Where information was not reported in the primary source, cells are marked NR. See also Figure 2.

## Abbreviations

The following abbreviations are used in this manuscript:

General/Introduction	
iPSC	induced pluripotent stem cell
NMS	non-model species
OSKM	OCT4, SOX2, KLF4, c-MYC
ESC	embryonic stem cell
EpiSC	epiblast stem cell
MEF	mouse embryonic fibroblast
Section 2—Technical	
2i/LIF	two-inhibitor/leukaemia inhibitory factor
LIF	leukaemia inhibitory factor
FGF	fibroblast growth factor
TGF-β	transforming growth factor beta
JAK	Janus kinase
STAT3	signal transducer and activator of transcription 3
GSK3	glycogen synthase kinase 3
MEK	mitogen-activated extracellular signal-regulated kinase
VPA	valproic acid
saRNA	self-amplifying RNA
RT-PCR	reverse transcription polymerase chain reaction
CGH	comparative genomic hybridisation
ISSCR	International Society for Stem Cell Research
QC	quality control
shRNA	short hairpin RNA
KDM4A	lysine demethylase 4A
VPR	VP64-p65-Rta transcriptional activator
iCD	induced chemically defined
hPSC	human pluripotent stem cell
AMCHEPRY	(raguneprocel; trade name, no abbreviation needed)
IND	investigational new drug
GMP	good manufacturing practice
MHC	major histocompatibility complex
HLA	human leucocyte antigen
Section 3—Conservation	
ART	assisted reproductive technologies
SCNT	somatic cell nuclear transfer
iSCNT	interspecies somatic cell nuclear transfer
IVF	in vitro fertilisation
ICSI	intracytoplasmic sperm injection
IVG	in vitro gametogenesis
PGCLC	primordial germ cell-like cell
PGC	primordial germ cell
NWR	northern white rhinoceros
CRISPR	clustered regularly interspaced short palindromic repeats
Section 4—Veterinary and Comparative Medicine	
E-iPSC	equine iPSC
ciPSC	canine iPSC
fiPSC	feline iPSC
piPSC	porcine iPSC
biPSC	bovine iPSC
DCM	dilated cardiomyopathy
HCM	hypertrophic cardiomyopathy
DMD	dystrophin gene
RPE	retinal pigment epithelium
MSC	mesenchymal stem cell
ASFV	African swine fever virus
PRRSV	porcine reproductive and respiratory syndrome virus
SLA-DM	swine leucocyte antigen DM
CD163	cluster of differentiation 163
FIPV	feline infectious peritonitis virus
FIV	feline immunodeficiency virus
ADE	antibody-dependent enhancement
SARS-CoV-2	severe acute respiratory syndrome coronavirus 2
ACE2	angiotensin-converting enzyme 2
WHO	World Health Organisation
NHP	non-human primate
PSEN1	presenilin 1
MYBPC3	myosin binding protein C3
NPC1	Niemann-Pick type C1
Section 5—Agriculture	
iTPSC	induced totipotent stem cell
SV40LT	simian virus 40 large T antigen
GGTA1	galactosyltransferase alpha 1
CMAH	cytidine monophospho-N-acetylneuraminic acid hydroxylase
B4GalNT2	beta-1,4-N-acetylgalactosaminyltransferase 2
FDA	Food and Drug Administration
USDA	United States Department of Agriculture
Section 6—Environmental Protection	
OECD	Organisation for Economic Co-operation and Development
AHR	aryl hydrocarbon receptor
ARNT	AHR nuclear translocator
CYP1A1	cytochrome P450 1A1
CYP1B1	cytochrome P450 1B1
PAH	polycyclic aromatic hydrocarbon
PCB	polychlorinated biphenyl
EDC	endocrine-disrupting chemical
ERα	oestrogen receptor alpha
AR	androgen receptor
TR	thyroid receptor
NRF2	nuclear factor erythroid 2-related factor 2
KEAP1	kelch-like ECH-associated protein 1
ROS	reactive oxygen species
WNT	wingless-related integration site
BMP	bone morphogenetic protein
NODAL	nodal growth differentiation factor
ACTIVIN	activin A
