# Supplementary material for: Induced Pluripotent Stem Cells in Non-Model Species: Applications and Challenges
Source: Cells. 2026 Aug 28;15(17):1565. doi: 10.3390/cells15171565 (PMC13564788; doi:10.3390/cells15171565)
Supplement: Supplementary file 1 [file cells-15-01565-s001.zip › cells-4514737-supplementary.pdf]

Year	Species; Cell source	Reprogramming method	Integration status	Pluripotency validation	Differentiation demonstrated & major application
2011	Drill (*Mandrillus leucophaeus*) & Northern white rhinoceros (*Ceratotherium simum cottoni*); Skin fibroblasts	Retroviral vectors; NR (Yamanaka factors implied)	Integrating retroviral	AP activity; OCT4, SOX2, NANOG, SSEA-4, TRA-1-60 expression; EB formation	EB tri-lineage differentiation reported. Conservation - first iPSCs from endangered species [7]
2012	Snow leopard (*Panthera uncia*); Skin fibroblasts	Moloney-based pMXs retroviral vectors; OSKM	Integrating retroviral	AP activity; OCT4, SOX2, NANOG expression; EB formation; NANOG critical for colony stabilisation	EB tri-lineage. Conservation - felid iPSC derivation [65]
2015	Captive orangutan (*Pongo pygmaeus*); Skin fibroblasts	Retroviral vectors; OSKM	Integrating retroviral	AP activity; pluripotency marker expression; EB formation	EB tri-lineage. Conservation - derivation from cryopreserved cells [66]
2017	Ryukyu spiny rat (*Tokudaia osimensis*); Tail fibroblasts	PiggyBac transposon, doxycycline-inducible; OSKM + NANOG	Mixed - excisable transposon	AP activity; pluripotency marker expression; interspecific chimera formation with mice confirmed	Germline-competent chimeras in mice; spermatogonia from female iPSCs. Conservation - IVG proof-of-concept [67]
2018	Tasmanian devil (*Sarcophilus harrisii*); Dermal fibroblasts	Lentiviral vectors; OSKM + NANOG + LIN28	Integrating lentiviral	AP activity; pluripotency markers; EB formation; teratoma formation confirmed	EB tri-lineage; teratoma. Conservation - marsupial iPSC derivation [68]
2019	Platypus (*Ornithorhynchus anatinus*); Skin fibroblasts	Lentiviral vectors; OSKM + NANOG + LIN28	Integrating lentiviral	AP activity; pluripotency marker expression; EB formation	EB tri-lineage. Basic research - evolution of pluripotency in monotremes [69]

2021	Northern white rhinoceros (*Ceratotherium simum cottoni*), 9 individuals; Skin fibroblasts	Sendai virus; OSKM	Non-integrating Sendai virus	AP activity; OCT4, SOX2, NANOG, SSEA-4, TRA-1-60/1-81; EB formation; transgene silencing confirmed	PGCLCs induced; allelic diversity captured across 9 individuals. Conservation - functionally extinct species; IVG pipeline [9]
2022	Avian species: Okinawa rail (*Hypotaenidia okinawae*), Japanese ptarmigan (*Lagopus muta japonica*), Blakiston's fish owl (*Bubo blakistoni*), Japanese golden eagle (*Aquila chrysaetos japonica*); Pinfeather pulp cells	PiggyBac transposon; M3O, Sox2, Klf4, c-Myc, Nanog, Lin28, Klf2	Mixed - excisable transposon	POU5F3, SOX2/SOX3, NANOG expression; AP activity; EB formation; chimera contribution in chicken embryos (ptarmigan)	Interspecific chimeras; germline competence not observed. Conservation - first avian endangered species iPSCs [60]
2022	Grevy's zebra (*Equus grevyi*); Fibroblasts	Retroviral vectors; OSKM	Integrating retroviral	AP activity; pluripotency marker expression; EB formation	EB tri-lineage. Conservation - equid iPSC derivation [71]
2022	Sumatran rhinoceros (*Dicerorhinus sumatrensis*), last male Kertam; Skin fibroblasts	Sendai virus (adapted from rhinoceros protocols); OSMK	Non-integrating Sendai virus	AP activity; OCT4, SOX2, NANOG expression; EB formation; transgene silencing confirmed	Differentiation to beating cardiomyocytes, neural progenitors, cerebral organoids. Conservation & Medicine - disease modelling; organoids [10]
2022	Fishing cat (*Prionailurus viverrinus*); Fibroblasts	PiggyBac transposon; episomal vectors; self-replicating RNA (multiple attempts); NR	Mixed - multiple approaches attempted	Intermediate cells obtained; no expandable bona fide iPSC colonies confirmed	No differentiation reported. Conservation - biobanking; reprogramming bottleneck documented [70]

2024	Giant panda (*Ailuropoda melanoleuca*); Fibroblasts	Episomal vectors + A83-01 (TGF-β inhibitor) + FACL chemically defined medium (iCD3 cocktail); OSK+ miRNA	Non-integrating episomal; chemically defined	AP activity; OCT4, SOX2, NANOG expression; EB formation; A83-01 increases efficiency >5-fold	EB tri-lineage. Conservation — fully chemically defined NMS reprogramming [61]
2024	SE Asian primates: Celebes crested macaque (*Macaca nigra*), Lar gibbon (*Hylobates lar*), Siamang (*Symphalangus syndactylus*), Proboscis monkey (*Nasalis larvatus*)	Sendai virus or episomal vectors; OSMK+species-specific formulations NR	Sendai virus or episomal	OCT4, SOX2, NANOG, SSEA-4, TRA-1-60 expression; AP activity; EB formation; transgene silencing confirmed	EB tri-lineage. Conservation — disease modelling; genetic safety nets [72]
2024	Bornean orangutan (*Pongo pygmaeus*); Fibroblasts or PBMCs	Sendai virus; OSKM	Non-integrating Sendai virus	AP activity; OCT4, SOX2, NANOG, SSEA-4, TRA-1-60/1-81; human-like naïve pluripotency confirmed by transcriptomics; transgene silencing confirmed	PGCLCs induced; potential for IVG pipeline. Conservation — naïve pluripotency; IVG [73]
2024	Bats (two species, not specified); NR	Sendai virus reprogramming kit; OSKM	Non-integrating Sendai virus	Pluripotency marker expression; AP activity; EB formation	No directed differentiation reported. Conservation — Basic research — viral tolerance; threatened bat preservation [74]

Supplementary Tabe S1. Timeline of iPSC derivation milestones across non-model species, 2011–2024, with cell source, reprogramming method, factors used, integration status, pluripotency validation assays reported, differentiation demonstrated, and major application domain for each species entry. Where information was not reported in the primary source, cells are marked NR. *See also Figure 2*
